# Development and evaluation of a secondary reference panel for *BCR-ABL1* quantification on the International Scale

**DOI:** 10.1038/leu.2016.90

**Published:** 2016-06-03

**Authors:** N C P Cross, H E White, T Ernst, L Welden, C Dietz, G Saglio, F-X Mahon, C C Wong, D Zheng, S Wong, S-S Wang, S Akiki, F Albano, H Andrikovics, J Anwar, G Balatzenko, I Bendit, J Beveridge, N Boeckx, N Cerveira, S-M Cheng, D Colomer, S Czurda, F Daraio, S Dulucq, L Eggen, H El Housni, G Gerrard, M Gniot, B Izzo, D Jacquin, J J W M Janssen, S Jeromin, T Jurcek, D-W Kim, K Machova-Polakova, J Martinez-Lopez, M McBean, S Mesanovic, G Mitterbauer-Hohendanner, H Mobtaker, M-J Mozziconacci, T Pajič, N Pallisgaard, P Panagiotidis, R D Press, Y-Z Qin, J Radich, T Sacha, T Touloumenidou, P Waits, E Wilkinson, R Zadro, M C Müller, A Hochhaus, S Branford

**Affiliations:** 1 Wessex Regional Genetics Laboratory, Salisbury NHS Foundation Trust, Salisbury, UK; 2 Faculty of Medicine, University of Southampton, Southampton, UK; 3 Department of Hematology/Oncology, Universitätsklinikum Jena, Jena, Germany; 4 Department of Genetics and Molecular Pathology, Centre for Cancer Biology, SA Pathology, Adelaide, SA, Australia; 5 III. Medizinische Klinik, Medizinische Fakultät Mannheim, Universität Heidelberg, Mannheim, Germany; 6 Department of Clinical and Biological Sciences, San Luigi Hospital, University of Turin, Orbassano, Italy; 7 Bergonie Institute Cancer Center Bordeaux, INSERM U1218, University of Bordeaux, Bordeaux, France; 8 Novartis Pharmaceuticals Corporation, East Hanover, NJ, USA; 9 West Midlands Regional Genetics Laboratory, Birmingham, UK; 10 Department of Hematology, University of Bari, Bari, Italy; 11 Laboratory of Molecular Diagnostics, Hungarian National Blood Transfusion Service, Budapest, Hungary; 12 Department of Pathophysiology, Semmelweis University, Budapest, Hungary; 13 King's College Hospital London, London, UK; 14 National Specialized Hospital for Active Treatment of Hematological Diseases, Sofia, Bulgaria; 15 Laboratorio de Biologia Tumoral, Disciplina de Hematologia do HC-FMUSP, São Paulo, Brazil; 16 PathWest Laboratory Medicine WA, Department of Haematology, Fiona Stanley Hospital, Perth, WA, Australia; 17 Department of Laboratory Medicine, University Hospitals Leuven, Leuven, Belgium; 18 Department of Oncology, KUL, Leuven, Belgium; 19 Department of Genetics, Portuguese Oncology Institute, Porto, Portugal; 20 Department of Hematology and Oncology, Quest Diagnostics Nichols Institute, San Juan Capistrano, CA, USA; 21 Hematopathology Unit, Hospital Clinic, IDIBAPS, Barcelona, Spain; 22 Division of Molecular Microbiology, Children's Cancer Research Institute, Vienna, Austria; 23 Department of Clinical and Biological Sciences, San Luigi Hospital, University of Turin, Orbassano, Italy; 24 Laboratoire Hematologie, Centre Hospitalier Universitaire de Bordeaux, Universite Bordeaux, Bordeaux, France; 25 Laboratory of Molecular Pathology, Oslo University Hospital, Oslo, Norway; 26 Clinique de Genetique Oncologique-Service de genetique, Hopital Erasme, Brussels, Belgium; 27 Imperial Molecular Pathology, Hammersmith Hospital, London, UK; 28 Department of Hematology and Bone Marrow Transplantation, Poznan University of Medical Sciences, Poznan, Poland; 29 Department of Clinical Medicine and Surgery, University ‘Federico II' of Naples, Naples, Italy; 30 CEINGE – Biotecnologie Avanzate, Naples, Italy; 31 Genoptix, Inc., Carlsbad, CA, USA; 32 Department of Hematology and Molecular Diagnostics, VU University Medical Center, Amsterdam, The Netherlands; 33 MLL Munich Leukemia Laboratory, Munich, Germany; 34 Center of Molecular Biology and Gene Therapy, Department of Internal Medicine-Hematology and Oncology, Masaryk University and University Hospital Brno, Brno, Czech Republic; 35 Seoul St Mary's Hospital, Leukemia Research Institute, The Catholic University of Korea, Seoul, Korea; 36 Department of Molecular Genetics, Institute of Hematology and Blood Transfusion, Prague, Czech Republic; 37 Department of Hematology, Hospital Universitario 12 de Octubre, Universidad Complutense, CNIO, Madrid, Spain; 38 Division of Cancer Medicine, Department of Pathology, Peter MacCallum Cancer Centre, East Melbourne, VIC, Australia; 39 Pathology Department, University Clinical Center Tuzla, Tuzla, Bosnia and Herzegovina; 40 Department of Laboratory Medicine, Division of Medical and Chemical Laboratory Diagnostics, Medical University of Vienna, Vienna, Austria; 41 Departement de Biopathologie, Institut Paoli-Calmettes, Marseille, France; 42 Specialized Haematology Laboratory, Department of Haematology, University Medical Centre Ljubljana, Ljubljana, Slovenia; 43 Department of Surgical Pathology, Zealand University Hospital, Roskilde, Denmark; 44 Hematology Unit, First Department of Internal Medicine, Laiko Hospital, University of Athens, Athens, Greece; 45 Department of Pathology and Knight Cancer Institute, Oregon Health and Science University, Portland, OR, USA; 46 Peking University People's Hospital, Peking University Institute of Hematology, Beijing, China; 47 Fred Hutchinson Cancer Research Center, Seattle, WA, USA; 48 Chair and Department of Hematology, Jagiellonian University, Kraków, Poland; 49 Laboratory of Molecular Biology, Hematology Department and HCT Unit, G. Papanicolaou Hospital, Thessaloniki, Greece; 50 Bristol Genetics Laboratory, Bristol, UK; 51 HMDS, Leeds Teaching Hospitals, Leeds, UK; 52 Faculty of Pharmacy and Biochemistry and University Hospital Center Zagreb, University of Zagreb, Zagreb, Croatia; 53 School of Pharmacy and Medical Science, University of South Australia, Adelaide, SA, Australia; 54 School of Medicine, University of Adelaide, SA, Adelaide, Australia; 55 School of Molecular and Biomedical Science, University of Adelaide, Adelaide, SA, Australia

## Abstract

Molecular monitoring of chronic myeloid leukemia patients using robust *BCR-ABL1* tests standardized to the International Scale (IS) is key to proper disease management, especially when treatment cessation is considered. Most laboratories currently use a time-consuming sample exchange process with reference laboratories for IS calibration. A World Health Organization (WHO) *BCR-ABL1* reference panel was developed (MR^1^–MR^4^), but access to the material is limited. In this study, we describe the development of the first cell-based secondary reference panel that is traceable to and faithfully replicates the WHO panel, with an additional MR^4.5^ level. The secondary panel was calibrated to IS using digital PCR with *ABL1*, *BCR* and *GUSB* as reference genes and evaluated by 44 laboratories worldwide. Interestingly, we found that >40% of *BCR-ABL1* assays showed signs of inadequate optimization such as poor linearity and suboptimal PCR efficiency. Nonetheless, when optimized sample inputs were used, >60% demonstrated satisfactory IS accuracy, precision and/or MR^4.5^ sensitivity, and 58% obtained IS conversion factors from the secondary reference concordant with their current values. Correlation analysis indicated no significant alterations in %BCR-ABL1 results caused by different assay configurations. More assays achieved good precision and/or sensitivity than IS accuracy, indicating the need for better IS calibration mechanisms.

## Introduction

The development of *BCR-ABL1* tyrosine kinase inhibitors, from the first-generation imatinib to newer agents such as nilotinib and dasatinib, has enabled progressively deeper molecular responses in chronic myeloid leukemia (CML) patients undergoing tyrosine kinase inhibitor therapy.^1, 2^ Deeper molecular responses are defined as *BCR-ABL1* levels of ⩽0.01% (MR^4^) and ⩽0.0032% (MR^4.5^) on the international reporting scale (International Scale (IS)) and are important milestones for patients considering treatment cessation.^3^ Other landmarks on the IS also represent different treatment decision thresholds and prognostic outcomes.^4^ For example, patients who reach 10% IS or below at 3 months after treatment have significantly higher rates of MR^4.5^ by 5 years,^5^ and reaching 0.1% IS (major molecular response) by 12 months of treatment is predictive of subsequently achieving undetectable *BCR-ABL1* levels.^6^ Thus, regular molecular monitoring using real-time reverse-transcription quantitative PCR (RT-qPCR) is recommended for optimal disease management, and treatment decisions rely on achieving milestone molecular responses in the first year of therapy and beyond.^2, 7^

As treatment decisions are directly impacted by test results, accuracy and precision of *BCR-ABL1* assays across the entire measurement range is crucial for patient management, especially in patients with deep molecular responses when considering possible treatment cessation. It is well known that high variability exists between RT-qPCR methods used in different laboratories.^8, 9^ The first international standardization attempt occurred in 2003, when different *BCR-ABL1* assays used in the International Randomised Study of Interferon versus STI571 (IRIS) trial established IS based on 30 CML patient samples.^10^ Subsequently, a process for establishing a test-specific IS conversion factor (CF) by exchanging 20–30 CML patient samples with a reference laboratory was developed.^11^ Although this process works well for laboratories with tests that show good stability over time, it is time consuming, expensive and difficult to access for smaller laboratories.^12, 13^

In 2010, the ‘first International Genetic Reference Panel for quantitation of *BCR-ABL* mRNA' was developed as a primary standard for *BCR-ABL1* assay IS calibration and accredited by the World Health Organization (WHO).^14^ The WHO panel is made of lyophilized K562 and HL-60 cell line mixtures, which allows the inclusion of cellular RNA extraction in the IS calibration against the two major *BCR-ABL1* breakpoints (e13a2 and e14a2) and carries three sets of nominal %BCR-ABL1 values using *ABL1*, *BCR* and *GUSB* as reference genes. Owing to restricted access, the WHO panel is currently only available to manufacturers of *BCR-ABL1* test kits and secondary standards.^13^ The commercial secondary standards available to date are made of RNA;^15, 16^ thus, RNA extraction is not included in the IS calibration process, except when the standards are artificially spiked into the cell samples. Furthermore, none of these are calibrated to the WHO panel against all three reference genes.

In this study, we describe the successful development of the first cell-based *BCR-ABL1* secondary reference panel that is traceable to and faithfully replicates the WHO panel in both raw materials (lyophilized K562 and HL-60 cell mixes) and manufacturing process, with the addition of a MR^4.5^ level. Nominal %BCR-ABL1 IS values were assigned to the secondary panel using reverse-transcription droplet digital PCR (RT-ddPCR) against *ABL1*, *BCR* and *GUSB*. The secondary panel was successfully evaluated by 45 different *BCR-ABL1* assays in a subsequent international multi-center evaluation study.

## Materials and methods

### Manufacturing and IS calibration of secondary reference panel

K562 (ATCC CCL-243) and HL-60 cells (ATCC CCL-240) (American Type Culture Collection, Manassas, VA, USA) were cultured, mixed and lyophilized following methods described by White *et al.*^14^ with minor modifications (Supplementary Information). Calibration to the WHO standards was performed as described.^14^ IS calibration using *ABL1* as a reference gene was conducted using 10 sets of WHO ‘first International Genetic Reference Panel for quantitation of BCR-ABL mRNA' panels (National Institute for Biological Standards and Control, South Mimms, UK). Calibration using *BCR* and *GUSB* was conducted in a second study using another 10 sets of WHO panels. On each day of 10 non-consecutive days, 1 WHO panel and 2–3 secondary panels were tested using RT-ddPCR in 4 replicates for the MR^1^ (10% BCR-ABL^IS^) to MR^4^ (0.01% BCR-ABL^IS^) samples and in 8 replicates for the MR^4.5^ (0.0032% BCR-ABL^IS^) sample, to enhance assay precision. Data analysis was performed using the statistical methods described by White *et al.*^14^

### Reverse-transcription droplet digital PCR

RNA extraction from the secondary panel was performed using RNeasy mini kits (Qiagen, Hilden, Germany). Reverse transcription was performed using ABI High Capacity cDNA reverse-transcription kit (Thermo Fisher Scientific, Waltham, MA, USA) and ddPCR was performed using 2X ddPCR Supermix (Bio-Rad, Hercules, CA, USA) on the QX-100 or QX-200 ddPCR system (Bio-Rad). All primer and probe sequences are listed in Table 1. *BCR-ABL1* and reference genes were run as singleplex reactions in separate wells. To achieve optimal assay precision and avoid signal saturation, cDNA input for *BCR-ABL1* per 20 μl reaction was 80 ng for the MR^1^ sample, 400 ng for the MR^2^ sample and 675 ng for samples ⩽MR^3^. cDNA input for reference genes was 10 ng per 20 μl reaction for all three reference genes. For each RT-ddPCR run, wells with >9025 accepted droplets were considered valid as per the manufacturer's recommendations.

## Results

### Manufacturing and IS calibration of the WHO *BCR-ABL1* secondary reference panel

We successfully manufactured >12 000 vials of secondary *BCR-ABL1* lyophilized cell reference panel, using the same K562 and HL-60 cell lines and following similar manufacturing procedures as the primary WHO panel (Supplementary Information).^14^ An MR^4.5^ level was added to the secondary panel, to enable more accurate IS calibration at this critical level, as CML patients reaching this deep molecular response are increasingly being considered for treatment cessation. Quality-control assessments indicated that the secondary panel had minimal residual moisture, excellent vial-to-vial homogeneity and >2.5 years of real-time stability (Supplementary Information).

To calibrate the secondary panel to the WHO ‘first International Genetic Reference Panel for quantitation of *BCR-ABL1* mRNA', we followed the study design described by White *et al.*,^14^ except that the sample size was doubled to strengthen the statistical power (Supplementary Information). RT-ddPCR was chosen as the calibration method, owing to its superior sensitivity, precision and absolute quantification capability compared with RT-qPCR.^17, 18^ At the time of this study, no commercially available *BCR-ABL1* test used *BCR* or *GUSB* as reference genes. We developed three sets of RT-ddPCR assays, including *BCR-ABL1/ABL1*, *BCR-ABL1/BCR* and *BCR-ABL1/GUSB*, to enable IS calibration of the secondary panel against all three reference genes. All RT-ddPCR assays were validated following a combination of industry best practices, Minimum Information for Publication of Quantitative Real-Time PCR Experiments guideline, and Clinical and Laboratory Standards Institute guideline, to ensure proper accuracy, precision, sensitivity and linearity were achieved (Supplementary Information).^19, 20, 21, 22^ Using methods described by White *et al.*,^14^ we determined the IS CF for the RT-ddPCR assays to be 0.93 for *BCR-ABL1/ABL1*, 1.85 for *BCR-ABL1/BCR* and 1.28 for *BCR-ABL1/GUSB*.

**Figure 1:**
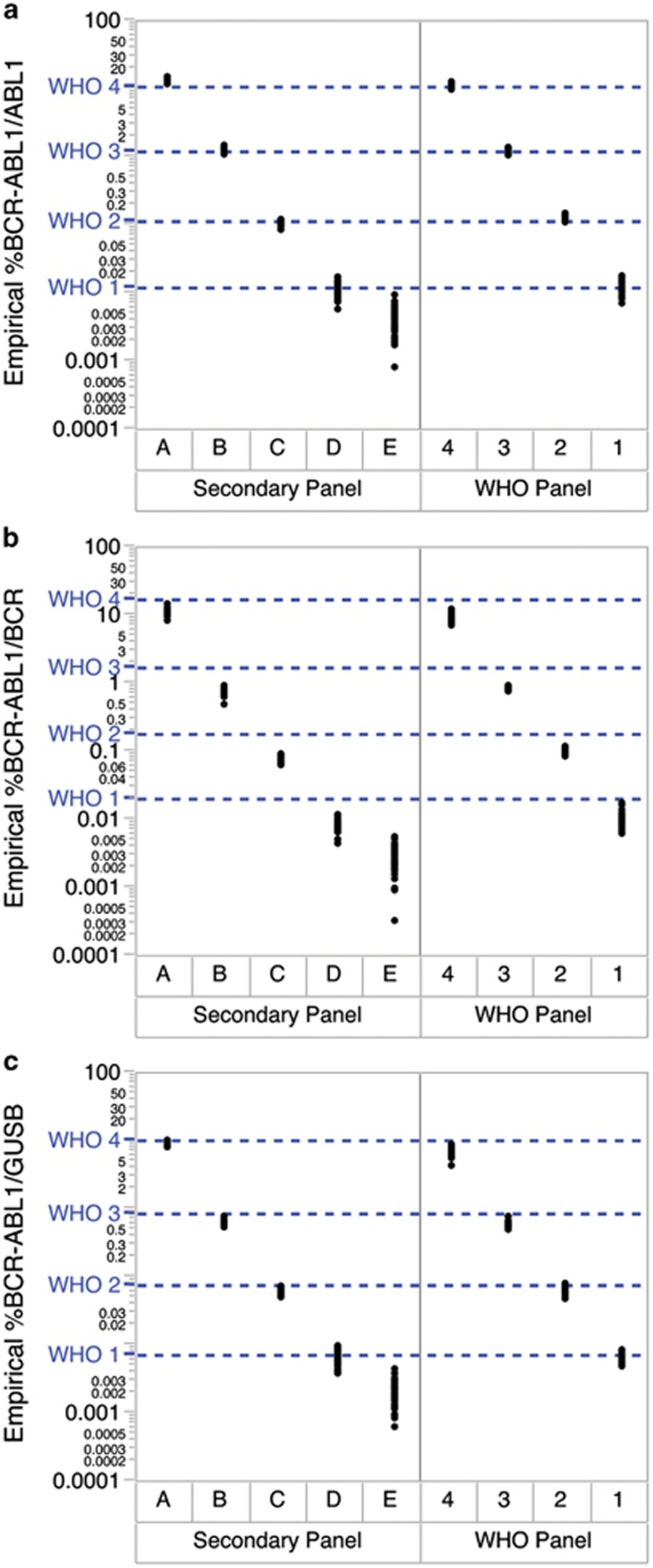
The *BCR-ABL1* secondary reference panel was calibrated to the WHO standards using RT-ddPCR against (**a**) *ABL1*, (**b**) *BCR* and (**c**) *GUSB* for IS conversion (*n*=40 from MR^1^ to MR^4^, *n*=80 for MR^4.5^). The IS CFs for the RT-ddPCR assays were determined to be 0.93 for the *BCR-ABL1/ABL1* assay, 1.85 for the *BCR-ABL1/BCR* assay and 1.28 for the *BCR-ABL1/GUSB* assay. Blue dotted lines represent the nominal %BCR-ABL1 value of the WHO panel at different levels.

Each CF was subsequently applied to the empirical %BCR-ABL1 of the secondary panel measured by RT-ddPCR, to obtain the assigned %BCR-ABL1^IS^ values (Figure 1 and Table 2a). We found that the mean %BCR-ABL1 for each level of the secondary panel met all targeted *BCR-ABL1* levels and were within 1.3-fold of the WHO standards values. The assigned %BCR-ABL1^IS^ of level E was 0.0038%, 0.0050% and 0.0029% for *ABL1*, *BCR* and *GUSB*, respectively, indicating that an MR^4.5^ level was successfully created. Moreover, the mean copy number of *BCR-ABL1*, *ABL1*, *BCR* and *GUSB* per ng of RNA measured using RT-ddPCR was highly similar between the WHO and secondary panels (Table 2b). This demonstrated that the secondary panel replicated the primary WHO panel faithfully, with the successful addition of an MR^4.5^ level.

### Laboratory evaluation of the WHO *BCR-ABL1* secondary reference panel

#### Study design

**Figure 2:**
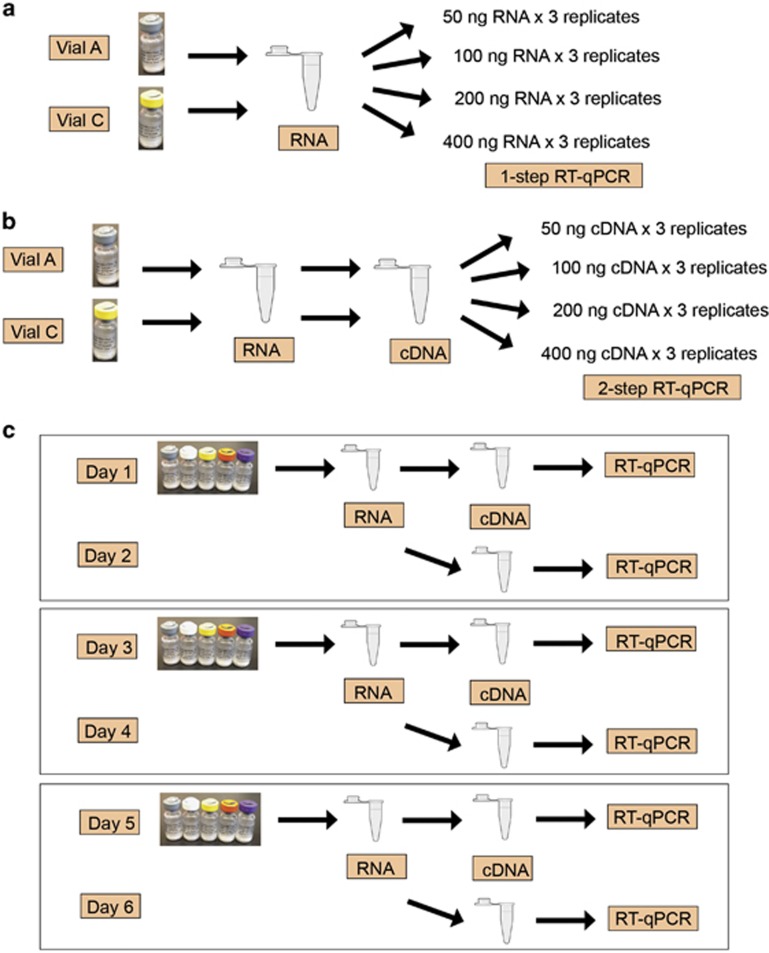
Study design for the international multi-center evaluation of the secondary panel, including (**a**) Study 1 for one-step RT-qPCR tests, (**b**) Study 1 for two-step RT-qPCR tests and (**c**) Study 2.

The secondary panel was sent to 44 clinical laboratories from 24 countries worldwide for evaluation, including 34 laboratories from Europe (Supplementary Table 2). One laboratory tested the panel with two different *BCR-ABL1* assays, resulting in a total of 45 *BCR-ABL1* tests included in this report. The laboratories were asked to conduct two studies with the secondary panel. In Study 1, to determine the optimal sample input of the secondary panel specific for each *BCR-ABL1* test, a standard curve experiment was run with Vial A (MR^1^) and Vial C (MR^3^) of the panel using 50, 100, 200 and 400 ng of RNA (for one-step assays) or cDNA (for two-step assays) input per PCR reaction; three replicates were run per sample at each input level (Figures 2a and b). In Study 2, to assess the usability of the secondary panel and performance of the *BCR-ABL1* tests, laboratories used the optimal sample input determined in Study 1 to test three sets of the panel on six different days (Figure 2c).

#### Study 1: sample input optimization

**Figure 3:**
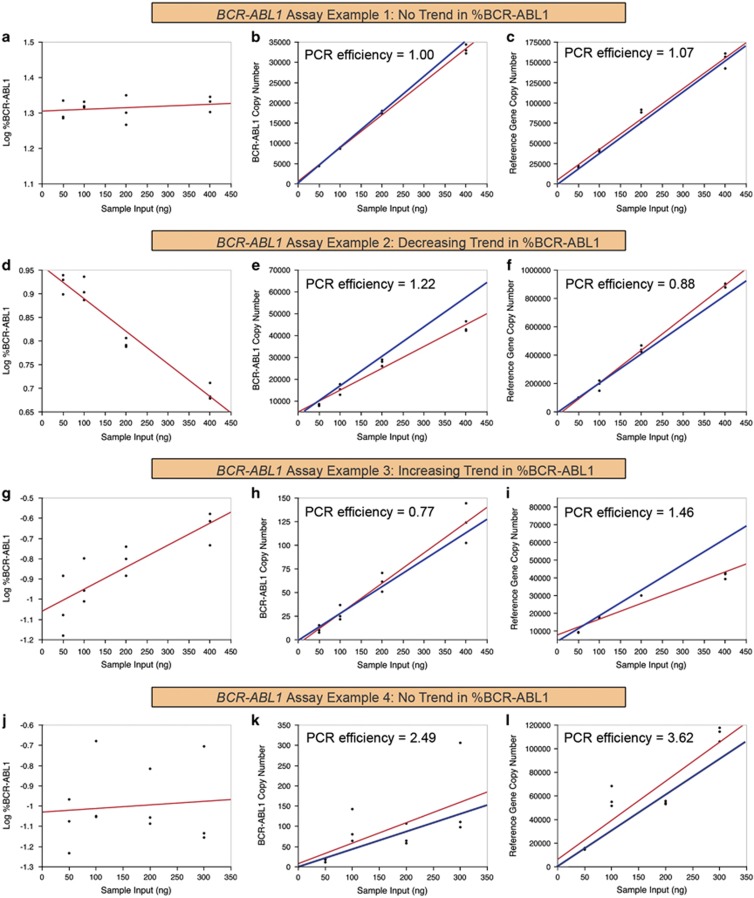
Examples of different assay results from Study 1 of the multi-center evaluation study. A properly optimized assay should yield similar %BCR-ABL1 results at different sample inputs and both the *BCR-ABL1* and reference gene assays should have a PCR efficiency close to 1 (**a**–**c**). Some assays showed decreasing %BCR-ABL1 measurements with increasing sample input (**d**–**f**), whereas others showed increasing %BCR-ABL1 measurements instead (**g**–**i**). The PCR efficiency of these assays tended to be suboptimal (<0.9 or >1.1), resulting in disproportional increase of *BCR-ABL1* or reference gene copy number with increasing sample input (**e**, **f**, **h** and **i**), which subsequently led to the unstable %BCR-ABL1 measurements (**d** and **g**). Occasionally, *BCR-ABL1* and reference gene assays that were suboptimal in a similar manner could cancel each other's defects, to achieve artificially stable %BCR-ABL1 measurements (**j**–**l**). Red lines represent the linear regression fit based on actual data and blue lines represent what ideal data should resemble.

The WHO panel did not offer recommendations on sample input for IS calibration. Nonetheless, sample input outside of the linear dynamic range of a RT-qPCR assay might potentially lead to inaccurate results. Thus, we designed a standard curve experiment to help laboratories determine the optimal sample input of the secondary panel for their *BCR-ABL1* tests (Figures 2a and b). Surprisingly, approximately half of the tests showed different %BCR-ABL1 results against different sample inputs of the same sample (*P*<0.05), even when data from either the highest or lowest sample input were allowed to be removed based on auxiliary-pick-regression analysis (Figure 3 and Supplementary Information). Among the 42 assays that tested Vial A (MR^1^), 29% (12 of 42) showed decreasing %BCR-ABL1 with increasing sample input (Figure 3d) and 24% (10 of 42) showed increasing %BCR-ABL1 (Figure 3g and Supplementary Figure 2). Among the 45 assays that tested Vial C (MR^3^), 22% (10 of 45) showed decreasing %BCR-ABL1 with increasing sample input (Figure 3d) and 18% (8 of 45) showed increasing %BCR-ABL1 (Figure 3g and Supplementary Figure 3).

In this study, we observed that the mean standard deviation (s.d.) in %BCR-ABL1 measurements from all 45 assays was 0.2log, which was mathematically equivalent to a 1.6-fold difference in the linear scale. Based on recommendations by Thiers *et al.*,^23^ 1 s.d. (0.2log) was considered the optimal cutoff value for determining differences in measurements in this study. Selecting 1 s.d. as the cutoff value took into consideration the fact that a <1-s.d. cutoff value would require a substantially larger sample size to be considered statistically robust, whereas >1 s.d. would increase the number of misclassifications.^23^ Thus, in this study, a mean difference of ⩾0.2log in %BCR-ABL1 value at different sample inputs by the same test was considered as beyond the inherent variability of an assay. We found that among the assays that showed changing %BCR-ABL1 against different sample inputs, 55% (12 of 22) at MR^1^ and 78% (14 of 18) at MR^3^ obtained results with ⩾0.2log difference. Overall, these results showed that some *BCR-ABL1* tests were nonlinear and might therefore yield statistically different %BCR-ABL1 results against different sample inputs. To mitigate the risk of inaccurate %BCR-ABL1 measurements from using an inappropriate sample input, it is highly recommended that laboratories standardize CML patient sample inputs by quantifying the extracted RNA before performing RT-qPCR.

To further investigate the cause of the unstable %BCR-ABL1 measurements across different sample inputs, we calculated the PCR efficiency and efficiency ratio for each individual *BCR-ABL1* and reference gene assay using the formula ‘Efficiency=−1+10^(−1/slope)^' (Supplementary Information).^24^ A well-optimized PCR assay should have PCR efficiency between 0.9 and 1.1,^25^ which would result in a PCR efficiency ratio of ~1 between the *BCR-ABL1* and reference gene assays. Indeed, we found that most assays that successfully achieved stable %BCR-ABL1 across different sample inputs had a PCR efficiency close to 1 for both the *BCR-ABL1* and reference gene assays, resulting in a mean efficiency ratio of 1.03 (*n*=43; abnormal efficiency ratios of <0 and >10 were excluded from the analysis) (Figures 3a–c). Assays that showed decreasing %BCR-ABL1 against increasing sample input had a mean PCR efficiency ratio of 1.51 (*n*=20) (Figures 3d–f), whereas assays that showed increasing %BCR-ABL1 had a mean PCR efficiency ratio of 0.63 (*n*=16) (Figures 3g–i). This indicated that the lack of stability in %BCR-ABL1 against sample input was directly correlated with suboptimal PCR efficiency. Surprisingly, *BCR-ABL1* and reference gene assays that were suboptimal in a similar manner could artificially cancel each other's defects to achieve artificially stable %BCR-ABL1 results (Figures 3j–l). Overall, these results illustrated that both the *BCR-ABL1* and reference gene assays needed to be properly optimized and validated, in order to achieve good quality %BCR-ABL1 testing.^26, 27, 28^

As different assays had different linear dynamic ranges, we observed a >30-fold range of optimal sample input for the secondary panel calculated for the different *BCR-ABL1* tests. Interestingly, among the 31 laboratories that routinely quantified their CML patient sample inputs, the reported patient sample input was on average 2.4-fold higher than the calculated optimal input of the secondary panel, after two extreme outlier values of 11.6- and 48.3-fold were identified using robust regression analysis and excluded from the calculation (Supplementary Information). This was concordant with the fact that although the mean copy number of *ABL1*, *BCR* and *GUSB* per ng of RNA in the secondary panel was 674, 1028 and 1245 (Table 2b), the mean copy per ng of human EDTA anticoagulated blood RNA was only 289 for *ABL1* (*n*=40), 750 for *BCR* (*n*=6) and 420 for *GUSB* (*n*=5) (data not shown). These results indicated that the optimal sample input of the secondary panel was approximately half of the patient sample input typically used by the laboratory in terms of ng RNA or cDNA. Thus, when using the secondary panel, laboratories might consider using twofold less cDNA input per PCR reaction compared with CML patient samples, to achieve similar copy numbers and avoid exceeding the linear dynamic range of their assay. Nonetheless, it is recommended that laboratories perform a similar standard curve experiment to identify the optimal sample input specific for their assay before using the secondary panel for the first time. The optimized sample input should maximize copy number detection but minimize potential PCR inhibition caused by carryover from the reverse-transcription reactions.

#### Study 2: performance of clinical BCR-ABL1 tests on the secondary panel

A robust *BCR-ABL1* test should demonstrate good IS accuracy, precision and sensitivity within statistical limits, especially at the lower disease levels. In Study 2, laboratories were asked to use the optimal sample input determined in Study 1 to test three sets of the secondary panel on six different days, following the design previously used by White *et al.* (Figure 2c).^14^ Results from each assay were subsequently analyzed to assess accuracy, precision and sensitivity. For IS accuracy, the overall mean %BCR-ABL1 from all 45 assays, calculated using robust regression analysis to minimize effects of outliers, were highly concordant with the assigned values of the secondary panel (Table 3 and Supplementary Figure 5). The mean ratios for all reference genes at all levels were within 1.5-fold or 0.16log of the panel's assigned values, with the exception of Vial D (MR^4^) for *GUSB*, which differed by 1.7-fold/0.24log. For results from individual assays, 60% (27 of 45) obtained mean %BCR-ABL1 within twofold of the panel's assigned values at all levels (Supplementary Figure 5). The observed concordance was most likely due to three factors. First, optimal sample input calculated in Study 1 was used for each assay, thus restricting the PCR reactions within the assay's linear dynamic range. Second, even though many different assay configurations were used by the laboratories (Supplementary Table 2), 60% (27 of 45) followed the EAC recommendations for the PCR primer design^26^ and 18% (8 of 45) used commercial *BCR-ABL1* kits (Supplementary Table 2 and Supplementary Figure 5), which are generally well optimized and validated. Lastly, 51% (23 of 45) of assays were IS calibrated via sample exchange with the reference laboratory in Mannheim, Germany, and 20% (9 of 45) with Adelaide, Australia. This demonstrated that *BCR-ABL1* tests can be effectively harmonized by using the same PCR primer designs and by standardizing the IS calibration process. Thus, commercial availability of a common IS reference material could contribute to worldwide IS standardization.

Most clinical samples are typically run in only one or two replicates, which requires a high degree of assay precision to ensure accuracy of each of the final *BCR-ABL1* test results. To enable ⩾95% confidence for the true value of a CML patient sample to be within 0.5log on each side of the measured value, an s.d. of ⩽0.25log for the *BCR-ABL1* test is required. Accordingly, if a sample is measured at MR^4.5^, there will be ⩾95% confidence that the true value of the sample is not above MR^4^ or below MR^5^.^29^ We calculated the intra-lab s.d. for each *BCR-ABL1* test and noted that 84% (38 of 45) successfully achieved an s.d. of ⩽0.25 log from MR^1^ to MR^4^. For *BCR-ABL1* tests that obtained >0.25log s.d., precision may be improved by performing further assay optimization and increasing the number of replicates per patient sample.^28, 30^

For monitoring deep molecular response, good *BCR-ABL1* assay sensitivity and precision are key performance characteristics. In Study 2, we found that 93% (42 of 45) assays successfully detected all replicates at MR^4^ and 76% (34 of 45) detected all replicates at MR^4.5^. To further understand how sample input affected assay sensitivity and precision, we analyzed results from the 32 *ABL1* assays, as this provided the largest sample size. Among the *ABL1* assays, 56% (18 of 32) detected all replicates at MR^4.5^ and achieved ⩽0.25log s.d., 19% (6 of 32) detected all replicates but had >0.25log s.d. and 25% (8 of 32) assays had at least 1 undetected replicate. Logistic regression analysis showed a strong positive correlation between increased sample input and increased detection rate (*P*=0.001). In addition, the median *ABL1* copy number per PCR reaction was 98 202 for laboratories that achieved good detection rate and precision, 74 923 for those that achieved good detection rate but suboptimal precision and 29 717 for those that had undetected replicates at MR^4.5^, further illustrating that an increased sample input could improve the sensitivity and precision of MR^4.5^ detection.

The laboratories that participated in this study used diverse assay configurations for *BCR-ABL1* testing (Supplementary Table 2). To determine whether assay configurations affected assay performance in terms of IS accuracy, precision and sensitivity, we performed Bayesian average analysis and found no statistically significant relationship between assay performance versus choice of reference gene, RNA extraction method and the usage of commercial versus laboratory-developed tests. Interestingly, among the assays that successfully achieved good accuracy (within twofold of assigned values), good precision (⩽0.25log s.d.) and good MR^4.5^ sensitivity (no undetected replicate), 82% (14 of 17) showed stable %BCR-ABL1 measurements against sample inputs at MR^3^ in Study 1, compared with 46% (13 of 28) among assays with less optimal assay performance. This illustrated that although *BCR-ABL1* assays of different designs can perform equally well, proper PCR optimization is required to ensure good clinical performance. Interestingly, the number of assays that achieved good precision (84%) and MR^4.5^ sensitivity (76%) exceeded the number that achieved good IS accuracy (60%), indicating that there remains an unmet need for a simple and broadly available IS calibration mechanism.

#### Calculating WHO IS CF from the secondary panel

Using the assigned *BCR-ABL1* IS values of the secondary panel, it was possible to calculate an IS CF traceable to the WHO panel for each test. Before CF calculation, Bland–Altman analysis was performed for each assay as described by White *et al.*,^14^ to assess whether bias was linear across the %BCR-ABL1 range. Of all the assays, 69% (31 of 45) showed no statistically significant trend in the Bland–Altman analysis, indicating that a valid CF could be calculated for these assays and compared with the laboratory's current CF, most of which were obtained from sample exchange. Mathematically, when the ratio between the assay's current CF and the CF calculated from the secondary panel is <0.63 or >1.58, the resulting %BCR-ABL1 values will have a >0.2log difference (Supplementary Information). Among the 31 assays, 58% (18 of 31) achieved a CF ratio between 0.63 and 1.58, indicating that their current CF was equivalent to the CF from the secondary panel. This was concordant with the fact that 60% of assays obtained mean %BCR-ABL1 within twofold of the panel's assigned values. This also indicated that the secondary panel can be effectively used to obtain IS CFs equivalent to sample exchange.

## Discussions

In this study, we successfully created the first cell-based *BCR-ABL1* secondary reference panel that faithfully replicated the WHO *BCR-ABL1* International Genetic Reference Panel in both raw material and manufacturing methods,^14^ with an additional MR^4.5^ level. The secondary panel was manufactured under Good Manufacturing Practice and confirmed to have low residual moisture content, vial-to-vial homogeneity and >2.5-year stability. *BCR-ABL1* IS values traceable to the WHO panel were assigned to the secondary panel using RT-ddPCR against *ABL1*, *BCR* and *GUSB*. Both the assigned IS values and absolute copy numbers of the secondary panel were found to be highly concordant with the primary WHO standards.

Many of the 44 laboratories that participated in the secondary panel evaluation currently act as a national reference laboratory for their country. The panel was successfully processed by all laboratories, indicating that it is compatible with many different *BCR-ABL1* test configurations. Through a standard curve experiment, we found that close to half of the tests showed signs of inadequate PCR optimization such as poor linearity against different sample inputs and suboptimal PCR efficiency. Interestingly, when a customized optimal sample input was used, 60% (27 of 45) of assays achieved mean %BCR-ABL1 values within twofold of the panel's assigned values, 84% (38 of 45) achieved good precision (⩽ 0.25log s.d.) from MR^1^ to MR^4^ and 76% (34 of 45) achieved 100% detection rate down to MR^4.5^. Three factors probably contributed to these excellent results: usage of a validated optimized sample input specific to the assay, the fact that 78% (35 of 45) assays used either the EAC primer design or a commercial kit and that 71% (32 of 45) assays were IS calibrated via sample exchange with one of the two major international reference centers. This indicated that using published assay designs and a harmonized IS calibration approach may lead to *BCR-ABL1* test standardization. Nonetheless, we noted that the number of assays that achieved good precision and sensitivity exceeded the number that achieved good IS accuracy, indicating that there remains an unmet need for a simple and broadly available calibration mechanism, such as this secondary panel, to ensure IS accuracy is maintained in laboratories over time.

We also showed that different assay designs including different reference genes, RNA extraction methods and usage of commercial kits versus laboratory-developed tests did not affect assay performance. Nonetheless, better PCR optimization correlated with better assay performance and increased sample input improved detection rate and precision at MR^4.5^. In addition to being a reference sample for IS calibration,^14^ the secondary reference material and its derivatives could also be used in assay analytical validation and optimization. For example, it can be used as standardized samples in External Quality Assessment programs for proficiency testing,^31^ especially as nominal values (that is, correct answers) are available for each member of the panel. Second, it can become a source of positive control samples to be run alongside patient samples for quality assurance. This can become especially powerful when multiple laboratories participate in a peer group quality-control monitoring program, in which results from such positive control samples are compared and monitored regularly. Lastly, the MR^4.5^ sample in the reference panel can be used to validate the sensitivity and MR^4.5^ detection capability of an assay.

In conclusion, a secondary reference panel traceable to the WHO panel with an additional MR^4.5^ level can provide easier access to IS calibration, as well as act as a tool for assay optimization, validation and quality assurance.

## Figures and Tables

**Table 1:** Primer and probe sequences for the RT-ddPCR assays

*Gene*	*Primer/probe*	*Sequence (5′–3′)*
*BCR-ABL1*	Forward primer	CCGCTGACCATCAATAAGGAA
	FAM MGB probe	AAGCCCTTCAGCGGC
	Reverse primer	CTGAGGCTCAAAGTCAGATGCTACT
*ABL1*	Forward primer	ACCACTGACGTGCCTGAGATG
	FAM MGB probe	AGAGAGCGATCCTCTGG
	Reverse primer	GAGACACGGCAGGCTCATG
*BCR*	Forward primer	CACTCAGCCACTGGATTTAAGC
	FAM MGB probe	CCTGGAGGTGGATTC
	Reverse primer	CGCGTCTTTGCTTTATTCACAA
*GUSB*	Forward primer	ACGCAGAAAATACGTGGTTGG
	FAM MGB probe	CTCATTTGGAATTTTGCCGAT
	Reverse primer	GCCGAGTGAAGATCCCCTTT

Abbreviation: RT-ddPCR, reverse-transcription droplet digital PCR.

**Table 2:** The *BCR-ABL1* secondary reference panel was highly concordant with the WHO panel

*Reference gene*	*RT-ddPCR IS CF*	*WHO panel*			*Secondary panel*		
		*Panel member*	*Mean empirical %BCR-ABL1*	*Nominal %BCR-ABL1*^*IS*^	*Panel member*	*Mean empirical %BCR-ABL1*	*Assigned %BCR-ABL1*^*IS*^
*(a) Empirical and %BCR-ABL*^*IS*^ *values*							
* ABL1*	0.93	4 (08/198)	11.2250	10.7469	A	13.7178	12.7575
		3 (08/196)	1.2540	1.1672	B	1.2841	1.1942
		2 (08/194)	0.1260	0.1112	C	0.1095	0.1019
		1 (08/192)	0.0130	0.0118	D	0.0116	0.0108
					E	0.0041	0.0038
* BCR*	1.85	4 (08/198)	9.8135	16.3129	A	11.5502	21.3678
		3 (08/196)	0.8441	1.6627	B	0.7300	1.3505
		2 (08/194)	0.0988	0.1753	C	0.0759	0.1404
		1 (08/192)	0.0098	0.0195	D	0.0081	0.0150
					E	0.0027	0.0050
* GUSB*	1.28	4 (08/198)	6.5834	10.1235	A	9.1800	11.7504
		3 (08/196)	0.5924	0.8295	B	0.6219	0.7960
		2 (08/194)	0.0677	0.0749	C	0.0628	0.0804
		1 (08/192)	0.0065	0.0071	D	0.0068	0.0087
					E	0.0023	0.0029

*Reference gene*	*WHO panel*		*Secondary panel*	
	*Panel member*	*Mean copy number per ng RNA*	*Panel member*	*Mean copy number per ng RNA*
*(b) Copy numbers of BCR-ABL1, ABL1, BCR and GUSB per ng of RNA*				
* BCR-ABL1*	4 (08/198)	92.98	A	103.27
	3 (08/196)	9.75	B	8.56
	2 (08/194)	0.99	C	0.72
	1 (08/192)	0.10	D	0.08
			E	0.03
* ABL1*	4 (08/198)	831.74	A	754.55
	3 (08/196)	778.56	B	665.80
	2 (08/194)	784.95	C	651.95
	1 (08/192)	785.90	D	653.85
			E	642.71
* BCR*	4 (08/198)	948.59	A	973.44
	3 (08/196)	1036.91	B	1058.25
	2 (08/194)	1004.05	C	1012.51
	1 (08/192)	1033.21	D	1079.15
			E	1017.74
* GUSB*	4 (08/198)	1380.43	A	1198.94
	3 (08/196)	1454.12	B	1238.22
	2 (08/194)	1390.83	C	1261.53
	1 (08/192)	1388.23	D	1283.79
			E	1244.11

Abbreviations: CF, conversion factor; IS, International Scale; RT-ddPCR, reverse-transcription droplet digital PCR; WHO, World Health Organization.

**Table 3:** Summary results of the secondary panel international multi-center evaluation study

*Reference gene*	*Number of assays*	*Vial (WHO/secondary panel)*	*%BCR-ABL1*^*IS*^					
			*WHO panel* *Nominal*	*Secondary panel* *Assigned*	*Results from 45 BCR-ABL1 assays*			
					*Mean*	*s.d.*	*Minimum*	*Maximum*
*ABL1*	32	4 (08/198)/A	10.7469	12.7575	11.4450	8.2225	3.6127	37.4603
		3 (08/196)/B	1.1672	1.1942	1.1771	0.9994	0.4014	4.4109
		2 (08/194)/C	0.1112	0.1019	0.1013	0.0931	0.0362	0.4553
		1 (08/192)/D	0.0118	0.0108	0.0110	0.0232	0.0033	0.1323
		NA/E		0.0038	0.0038	0.0038	0.0013	0.0159
*BCR*	5	4 (08/198)/A	16.3129	21.3678	21.3999	12.3547	13.6781	42.5295
		3 (08/196)/B	1.6627	1.3505	1.7243	1.2662	1.0215	4.2655
		2 (08/194)/C	0.1753	0.1404	0.1615	0.0863	0.0881	0.3025
		1 (08/192)/D	0.0195	0.0150	0.0153	0.0105	0.0071	0.0302
		NA/E		0.0050	0.0056	0.0047	0.0028	0.0134
*GUSB*	8	4 (08/198)/A	10.1235	11.7504	8.9008	3.7900	4.5344	14.9359
		3 (08/196)/B	0.8295	0.7960	0.7346	0.4133	0.3514	1.5494
		2 (08/194)/C	0.0749	0.0804	0.0587	0.0351	0.0218	0.1149
		1 (08/192)/D	0.0071	0.0087	0.0050	0.0033	0.0010	0.0106
		NA/E		0.0029	0.0020	0.0018	0.0005	0.0054

Abbreviations: NA, not applicable; WHO, World Health Organization.

## References

[bib1] Baccarani M, Cortes J, Pane F, Niederwieser D, Saglio G, Apperley J et al. Chronic myeloid leukemia: an update of concepts and management recommendations of European LeukemiaNet. J Clin Oncol 2009; 27: 6041–6051. 19884523 10.1200/JCO.2009.25.0779 PMC4979100

[bib2] Baccarani M, Deininger MW, Rosti G, Hochhaus A, Soverini S, Apperley JF et al. European LeukemiaNet recommendations for the management of chronic myeloid leukemia: 2013. Blood 2013; 122: 872–884. 23803709 10.1182/blood-2013-05-501569 PMC4915804

[bib3] Hehlmann R, Muller MC, Lauseker M, Hanfstein B, Fabarius A, Schreiber A et al. Deep molecular response is reached by the majority of patients treated with imatinib, predicts survival, and is achieved more quickly by optimized high-dose imatinib: results from the randomized CML-study IV. J Clin Oncol 2014; 32: 415–423. 24297946 10.1200/JCO.2013.49.9020

[bib4] Marin D, Ibrahim AR, Lucas C, Gerrard G, Wang L, Szydlo RM et al. Assessment of BCR-ABL1 transcript levels at 3 months is the only requirement for predicting outcome for patients with chronic myeloid leukemia treated with tyrosine kinase inhibitors. J Clin Oncol 2012; 30: 232–238. 22067393 10.1200/JCO.2011.38.6565 PMC6366954

[bib5] Hughes TP, Saglio G, Kantarjian HM, Guilhot F, Niederwieser D, Rosti G et al. Early molecular response predicts outcomes in patients with chronic myeloid leukemia in chronic phase treated with frontline nilotinib or imatinib. Blood 2014; 123: 1353–1360. 24335106 10.1182/blood-2013-06-510396 PMC4624459

[bib6] Branford S, Seymour JF, Grigg A, Arthur C, Rudzki Z, Lynch K et al. BCR-ABL messenger RNA levels continue to decline in patients with chronic phase chronic myeloid leukemia treated with imatinib for more than 5 years and approximately half of all first-line treated patients have stable undetectable BCR-ABL using strict sensitivity criteria. Clin Cancer Res 2007; 13: 7080–7085. 18056186 10.1158/1078-0432.CCR-07-0844

[bib7] O'Brien S, Radich JP, Abboud CN, Akhtari M, Altman JK, Berman E et al. Chronic myelogenous leukemia, version 1.2015. J Natl Compr Canc Netw 2014; 12: 1590–1610. 25361806 10.6004/jnccn.2014.0159

[bib8] Muller MC, Erben P, Saglio G, Gottardi E, Nyvold CG, Schenk T et al. Harmonization of BCR-ABL mRNA quantification using a uniform multifunctional control plasmid in 37 international laboratories. Leukemia 2008; 22: 96–102. 17943168 10.1038/sj.leu.2404983

[bib9] Muller MC, Cross NC, Erben P, Schenk T, Hanfstein B, Ernst T et al. Harmonization of molecular monitoring of CML therapy in Europe. Leukemia 2009; 23: 1957–1963. 19710700 10.1038/leu.2009.168

[bib10] Hughes TP, Kaeda J, Branford S, Rudzki Z, Hochhaus A, Hensley ML et al. Frequency of major molecular responses to imatinib or interferon alfa plus cytarabine in newly diagnosed chronic myeloid leukemia. N Engl J Med 2003; 349: 1423–1432. 14534335 10.1056/NEJMoa030513

[bib11] Hughes T, Deininger M, Hochhaus A, Branford S, Radich J, Kaeda J et al. Monitoring CML patients responding to treatment with tyrosine kinase inhibitors: review and recommendations for harmonizing current methodology for detecting BCR-ABL transcripts and kinase domain mutations and for expressing results. Blood 2006; 108: 28–37. 16522812 10.1182/blood-2006-01-0092 PMC1895821

[bib12] Branford S, Fletcher L, Cross NC, Muller MC, Hochhaus A, Kim DW et al. Desirable performance characteristics for BCR-ABL measurement on an international reporting scale to allow consistent interpretation of individual patient response and comparison of response rates between clinical trials. Blood 2008; 112: 3330–3338. 18684859 10.1182/blood-2008-04-150680

[bib13] Cross NC, Hochhaus A, Muller MC. Molecular monitoring of chronic myeloid leukemia: principles and interlaboratory standardization. Ann Hematol 2015; 94 (Suppl 2): S219–S225. 25814088 10.1007/s00277-015-2315-1

[bib14] White HE, Matejtschuk P, Rigsby P, Gabert J, Lin F, Lynn Wang Y et al. Establishment of the first World Health Organization International Genetic Reference Panel for quantitation of BCR-ABL mRNA. Blood 2010; 116: e111–e117. 20720184 10.1182/blood-2010-06-291641

[bib15] Brown JT, Laosinchai-Wolf W, Hedges JB, Watt CD, Van Deerlin VM, Fletcher L et al. Establishment of a standardized multiplex assay with the analytical performance required for quantitative measurement of BCR-ABL1 on the international reporting scale. Blood Cancer J 2011; 1: e13. 22829126 10.1038/bcj.2011.10 PMC3255280

[bib16] ELITechGroup. Philadelphia P210 RNA reference package insert, 2013.

[bib17] Yang R, Paparini A, Monis P, Ryan U. Comparison of next-generation droplet digital PCR (ddPCR) with quantitative PCR (qPCR) for enumeration of Cryptosporidium oocysts in faecal samples. Int J Parasitol 2014; 44: 1105–1113. 25229177 10.1016/j.ijpara.2014.08.004

[bib18] Kim TG, Jeong SY, Cho KS. Comparison of droplet digital PCR and quantitative real-time PCR for examining population dynamics of bacteria in soil. Appl Microbiol Biotechnol 2014; 98: 6105–6113. 24831026 10.1007/s00253-014-5794-4

[bib19] Huggett JF, Foy CA, Benes V, Emslie K, Garson JA, Haynes R et al. The digital MIQE guidelines: minimum information for publication of quantitative digital PCR experiments. Clin Chem 2013; 59: 892–902. 23570709 10.1373/clinchem.2013.206375

[bib20] Clinical and Laboratory Standards InstituteEvaluation of Detection Capability for Clinical Laboratory Measurement Procedures; Approved Guideline, 2nd edn. Clinical and Laboratory Standards Institute: Wayne, PA, 2012.

[bib21] Clinical and Laboratory Standards InstituteEvaluation of Precision Performance of Quantitative Measurement Methods; Approved Guideline, 2nd edn. Clinical and Laboratory Standards Institute: Wayne, PA, 2004.

[bib22] Clinical and Laboratory Standards InstituteEvaluation of the Linearity of Quantitative Measurement Procedures: A Statistical Approach; Approved Guideline. Clinical and Laboratory Standards Institute: Wayne, PA, 2003.

[bib23] Thiers RE, Wu GT, Reed AH, Oliver LK. Sample stability: a suggested definition and method of determination. Clin Chem 1976; 22: 176–183. 1248118

[bib24] Rasmussen R. Quantification on the Light Cycler. Rapid Cycle Real-Time PCR. Springer, 2001; pp 21–34.

[bib25] Raymaekers M, Smets R, Maes B, Cartuyvels R. Checklist for optimization and validation of real-time PCR assays. J Clin Lab Anal 2009; 23: 145–151. 19455629 10.1002/jcla.20307 PMC6649018

[bib26] Gabert J, Beillard E, van der Velden VH, Bi W, Grimwade D, Pallisgaard N et al. Standardization and quality control studies of 'real-time' quantitative reverse transcriptase polymerase chain reaction of fusion gene transcripts for residual disease detection in leukemia - a Europe Against Cancer program. Leukemia 2003; 17: 2318–2357. 14562125 10.1038/sj.leu.2403135

[bib27] Branford S, Hughes T. Diagnosis and monitoring of chronic myeloid leukemia by qualitative and quantitative RT-PCR. Methods Mol Med 2006; 125: 69–92. 16502578 10.1385/1-59745-017-0:69

[bib28] Jennings LJ, Smith FA, Halling KC, Persons DL, Kamel-Reid S, Molecular Oncology Resource Committee of the College of American Pathologists. Design and analytic validation of BCR-ABL1 quantitative reverse transcription polymerase chain reaction assay for monitoring minimal residual disease. Arch Pathol Lab Med 2012; 136: 33–40. 22208485 10.5858/arpa.2011-0136-OA

[bib29] Qiagen Inc. Summary of Safety and Effectiveness Data (SSED) of Artus CMV RGQ MDx Kit. U.S. Food and Drug Administration, 2014.

[bib30] Cross NC, White HE, Colomer D, Ehrencrona H, Foroni L, Gottardi E et al. Laboratory recommendations for scoring deep molecular responses following treatment for chronic myeloid leukemia. Leukemia 2015; 29: 999–1003. 25652737 10.1038/leu.2015.29 PMC4430701

[bib31] Clark J, Jack A, Holden J, Barnett D, Reilly J. BCR-ABL1 Quantitation: To IS or not to IS, that is the question? Haematologica 2011; 96 (Suppl 2): S82.

