# Supplementary material for: Development and evaluation of a secondary reference panel for BCR-ABL1 quantification on the International Scale
Source: Leukemia. 2016 Jun 3;30(9):1844–52. doi: 10.1038/leu.2016.90 (PMC5240017; doi:10.1038/leu.2016.90)
Supplement: Supplementary Information [file leu201690x1.doc]

**SUPPLEMENTARY INFORMATION**

1. **Manufacturing of the secondary panel**
   1. Cells

K562 (ATCC CCL-243) and HL-60 cells (ATCC CCL-240) were purchased from American Type Culture Collection (ATCC, Manassas, Virginia, USA). K562 cells were cultured in RPMI-1640 (ATCC) with 10% Fetal Bovine Serum (ATCC) and 1X Penicillin/Streptomycin (Calbiochem, Billerica, Massachusetts, USA). HL-60 cells were cultured in IMDM (ATCC) with 20% Fetal Bovine Serum (ATCC) and 1X Penicillin/Streptomycin (Calbiochem). Scaled-up cell culture was performed using roller bottles (Corning, New York, New York, USA) under Good Manufacturing Practice (GMP) for the manufacture of the secondary panel, and using HYPERFlasks (Corning) for the manufacture of an additional lot of MR^4.5^ level. Both cell lines were passaged every 3 days to maintain a density of 1.0 x 10^5^ to 1.0 x 10^6^ viable cells/mL and > 90% viability. Cells were quantified during each passage using a Cellometer (Nexcelom, Lawrence, Massachusettes, USA) for the full secondary panel, or a Vi-CELL (Beckman Coulter, Jersey City, New Jersey, USA) for the additional MR^4.5^ lot. All cell lines were under passage 22 at the time of manufacturing. A total of 8 L of HL-60 and 500 mL of K562 cells were cultured for the full panel, whereas 2 L of HL-60 and 15 mL of K562 cells were cultured for the additional MR^4.5^ level.

- 1. Cell mixing and lyophilization

K562 and HL-60 cells were harvested and resuspended in 4°C phosphate buffered saline (LONZA, Basel, Switzerland) to a density of 1 million cells/mL and 2 million cells/mL respectively. The cell lines were mixed together at predetermined ratios to achieve targeted *BCR-ABL1* levels of 10%, 1%, 0.1%, 0.01%, and 0.0032% and transported to the lyophilization facility at 4°C.

For lyophilization, 0.5 g of cell mixture (~1 million cells) was dispensed into ~2 500 of 2 mL flint glass vials (West Pharmaceutical Services, Exton, Philadelphia, USA) per *BCR-ABL1* level while maintaining 4°C with gentle stirring. 13 mm flurotec Igloo stoppers (West Pharmaceutical Services) were partially inserted into the vials. The filled vials were then loaded onto lyophilizer shelves pre-cooled at -45°C over 125 min. A vacuum of 75 mT was applied, and the temperature was increased to -10°C over 60 min and held for 720 min. After the primary hold, the temperature was raised to 30°C over 180 min and held for 375 min. To ensure proper moisture removal, the temperature was adjusted to 25°C over 60 min and held for an additional 960 min. The total lyophilization time was approximately 41 hr. After lyophilization, the vials were backfilled with nitrogen gas, and the stoppers were fully inserted and sealed with 13 mm tear off crimps (West Pharmaceutical Services). A total of 12 223 vials of lyophilized cell were successfully manufactured under GMP, with approximately 2 450 vials at each of the targeted 10%, 1%, 0.1%, 0.01% and 0.0032% *BCR-ABL1* level. An additional 2 500 vials of targeted 0.0032% *BCR-ABL1* level were created separately to illustrate good reproducibility in the manufacturing of lyophilized mixed cells at MR^4.5^. The secondary panel was kept at -20°C for long term storage.

1. **Quality assessment of secondary panel**
   1. Residual moisture

Karl Fischer test was conducted to confirm that all vials tested contained < 2% water content, meeting the product release criteria for lyophilization.

- 1. Vial-to-vial homogeneity

A vial-to-vial homogeneity study was performed to determine whether the secondary panel was manufactured in a homogeneous manner. The study was conducted over 5 non-consecutive days by 2 different operators in order to capture the testing variability. On each testing day, RNA was extracted from 3 vials of each level of the secondary panel, and 2 separate reverse transcription droplet digital polymerase chain (RT-ddPCR) reactions were performed for each RNA sample to allow for the comparison between inter-vial and intra-vial variability. A total of 30 RT-ddPCR reactions (*BCR-ABL1* and *ABL1*) from 15 vials were performed for each level of the secondary panel. We found that the total variance was in general very small. The total variance increased with decreasing *BCR-ABL1* levels, most likely due to the typical increased assay variability at lower analyte concentration. We then compared the inter-vial variance to intra-vial variance at each level of the secondary panel and found that there was no significant difference between the two (Supplementary Table 1). This indicated that the secondary panel had excellent vial-to-vial homogeneity.

| Level | Target %BCR-ABL1/ABL1 Level | Mean Measured %BCR-ABL1/ABL1 (non-IS) | Inter-vial Variance (% of total variance) | Intra-vial Variance (% of total variance) | Total Variance |
| --- | --- | --- | --- | --- | --- |
| A (Lot 1) | 10% | 13.1067 | 0.0328 (57.89%) | 0.0286 (42.11%) | 0.0436 |
| B (Lot 1) | 1% | 1.2097 | 0.0220 (45.45%) | 0.0255 (54.55%) | 0.0332 |
| C (Lot 1) | 0.1% | 0.0941 | 0.0532 (71.79%) | 0.0333 (28.21%) | 0.0624 |
| D (Lot 1) | 0.01% | 0.0109 | 0.1795 (78.16%) | 0.0948 (21.84%) | 0.2030 |
| E (Lot 1) | 0.0032% | 0.0044 | 0.1332 (49.86%) | 0.1336 (50.14%) | 0.1884 |
| E (Lot 2) | 0.0032% | 0.0051 | 0.1620 (67.88%) | 0.1114 (32.12%) | 0.1965 |

Supplementary Table 1: Vial-to-vial homogeneity analysis of the secondary panel.

- 1. RNA yield

We observed a difference in RNA yield between the secondary and the World Health Organization (WHO) First International Genetic Reference Panel for Quantitation of *BCR-ABL1* mRNA, with the average RNA yield being 6.9 µg per vial for the secondary panel, and 19.7 µg per vial for the WHO panel. The secondary panel contained ~1 million cells per vial, whereas the WHO panel contained ~1.5 million cells per vial.^1^ Since we acquired the cell lines from different repository than the WHO panel, it was possible that differences existed between these cell lines contributed to the different RNA yield. Nonetheless, we noted that the copy numbers of *ABL1, BCR, BCR-ABL1*, and *GUS* per ng RNA of the secondary panel were highly concordant with the copy numbers of the WHO panel (Table 2b in main manuscript). Given that the lyophilized cells contained ~2.5-fold higher *ABL1* copy per ng RNA compared to CML blood samples, we believe the lower RNA yield in our panel actually better represent CML patient samples, and that the difference in RNA yield will not contribute to any difference in the functionality of the secondary panel.

- 1. Real-time stability

A real-time stability study was conducted to track the long term stability of the secondary panel stored at -20°C with time points taken at 3-month intervals. At each time point, RNA was extracted from 3 vials per panel member, and RT-ddPCR was conducted in 2 replicates using *ABL1* as the reference gene. The average empirical %BCR-ABL1 was compared to the results from previous time points. An equivalence test was performed in which a log difference of > 0.2 from the t = 0 results was considered to be non-equivalent. To date, over 2.5 years of stability data have been collected for the lyophilized cell panel. With the exception of 2 outlier measurements at MR^4.5^, most likely due to change of reagent lots, all other time points tested at all 5 *BCR-ABL1* levels were within 0.2 log of the t = 0 data (Supplementary Figure 1). These data indicated that the lyophilized cell panel has remained stable and unchanged over 2 years of storage at -20°C. A similar stability study was also performed for a previous lot of lyophilized cell panel and > 3.5 years of stability has been demonstrated (data not shown).

Supplementary Figure 1: No significant trend in %BCR-ABL1 was observed with the secondary panel over 2 years.


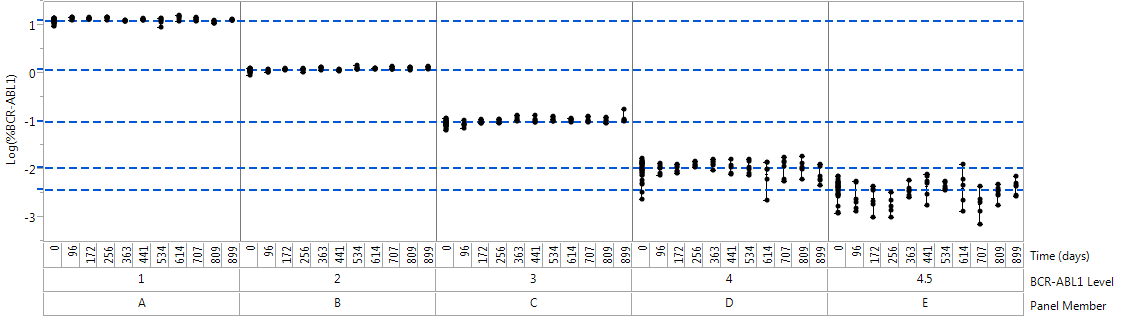


1. **International Scale calibration of the secondary panel**
   1. Methods

Twenty sets of the WHO panels were obtained from the National Institute for Biological Standards and Control (NIBSC, South Mimms, UK). International Scale (IS) calibration using *ABL1* as a reference gene was conducted first, using 10 sets of WHO panels, and calibration using *BCR* and *GUSB* was conducted in a second study, using another 10 sets of WHO panels. Both studies were completed in 10 non-consecutive days. On each day, 1 WHO panel and 2 - 3 secondary panels were tested using RT-ddPCR in 4 replicates for each sample from MR^1^ to MR^4^, and in 8 replicates for the MR^4.5^ samples to enhance assay precision. Data analysis was performed using the statistical methods described by White *et al.* ^1^ Due to instrument issues or operator errors, out of the 400 RT-ddPCR reactions in each study, 9 wells in *ABL1*, 4 wells in *BCR,* and 25 wells in *GUSB* were excluded from the analysis. Due to identification as an outlier by Grubb’ test, 0 well in *ABL1*, 7 wells in *BCR,* and 5 wells in *GUSB* were excluded.

- 1. Statistical justification for increasing the study design

Ten instead of 5 sets of WHO panel recommended by White *et al.*^1^ were used in the IS calibration of the secondary panel. The key parameter in this study is the estimated mean difference between the secondary panel and the WHO panel. The %BCR-ABL^IS^ value for each level of the secondary panel is assigned by adding/multiplying the anti-difference which is dependent on the scale of measure. As the secondary panel will be used as a secondary reference standard, any bias assigned to the secondary panel member will be forwarded into the newer assay, thereby resulting in bias of newer assay against the IS. Due to natural measurement variability, it is unavoidable to have some residual bias exist in the assigned IS value. We will control the magnitude of the residual bias by ensuring that sufficient number of replicates are performed. The first step is to determine the allowable magnitude of the residual bias. Thiers *et al.*^2^ proposed that a claim of sample chemistry stability is acceptable when the mean concentration is shown to change by less than an amount equal to 1 standard deviation (SD), with 5% risk of error. The same criteria will be applied to control the magnitude of the residual bias, where residual bias is to be less than or equal to 1 SD of the assay with 95% confidence. This criteria will require that the Margin of Error (MoE) of the estimation of mean difference between the WHO panel (X) and the secondary panel (Y) is less than or equal to
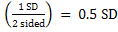
. Since the
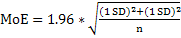
, it will require a sample size
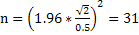
. For the secondary panel to function as an IS reference panel in the future, the lot-to-lot variation from the assays that will be calibrated using the secondary panel needs to be accounted for as well. In order to keep the total mean difference from lot-to-lot during the calibration of the secondary panel to less than 1 SD, an even smaller MoE is required for the estimation of mean difference between the WHO panel (X) and the secondary panel (Y). Assuming half of the MoE of 0.5 SD will be assignable to reagent lot-to-lot variability, 0.25 SD may be assessed as an appropriate MoE, which results in a sample size requirement of n = 124 (i.e.
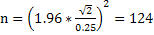
. Due to the fact that the WHO primary panel has 4 panel members, and the results can be pooled together to estimate the average difference, the sample size required from each of the 4 members will become 124/4= 41. In summary, 40 replicates for each of the 4 WHO panel members for this study are required. Thus this sample size is sufficient for this secondary panel to be used to calibrate for future manufacturing reagents which can allow for certain amount of lot-to-lot variability.

1. **Assay configurations of the 45 *BCR-ABL1* tests in the international multi-center evaluation study**

| Parameter | | Number of Labs | Percentage |
| --- | --- | --- | --- |
| Reference genes | *ABL* | 32 | 71.1% |
|  | *BCR* | 5 | 11.1% |
|  | *GUSB* | 8 | 17.8% |
| Assay type | Lab-developed test | 37 | 82.2% |
|  | Qiagen Ipsogen kit | 7 | 15.6% |
|  | ELITech kit | 1 | 2.2% |
| Reporting on International Scale | Yes | 42 | 93.3% |
|  | No | 3 | 6.7% |
| IS calibration method | Sample exchange only | 33 | 73.3% |
|  | Sample exchange plus another method | 5 | 11.1% |
|  | Commercial calibrators or kits | 4 | 8.9% |
|  | Not on IS | 3 | 6.7% |
| Reference lab for sample exchange | Mannheim | 23 | 51.1% |
|  | Adelaide | 9 | 20.0% |
|  | Other | 6 | 13.3% |
|  | Not on IS | 3 | 6.7% |
|  | Not applicable (Commercial calibrators or kits) | 4 | 8.9% |
| IS calibration frequency | Annually | 28 | 62.2% |
|  | Semi-annually | 5 | 11.1% |
|  | Quarterly | 1 | 2.2% |
|  | Other | 8 | 17.8% |
|  | Not on IS | 3 | 6.7% |
| RNA Extraction method | Trizol | 25 | 55.6% |
|  | Qiagen columns | 13 | 28.9% |
|  | MagNA Pure | 3 | 6.7% |
|  | Other | 4 | 8.9% |
| 1-step vs 2-step qRT-PCR | 2-step | 42 | 93.3% |
|  | 1-step | 3 | 6.7% |
| Reverse transcriptase | SuperScript | 26 | 57.8% |
|  | MMLV | 9 | 20.0% |
|  | MultiScribe | 6 | 13.3% |
|  | Others | 4 | 8.9% |
| Reverse transcription primers | Random primers | 42 | 93.3% |
|  | Gene-specific | 2 | 4.4% |
|  | Other | 1 | 2.2% |
| PCR instrument | ABI system | 25 | 55.6% |
|  | LightCycler | 15 | 33.3% |
|  | Rotor-gene | 5 | 11.1% |
| PCR Primer design | EAC | 27 | 60.0% |
|  | Other | 10 | 22.2% |
|  | Ipsogen kit | 7 | 15.6% |
|  | Nanogen kit | 1 | 2.2% |
| In-run calibrators | Ipsogen | 16 | 35.6% |
|  | ERM-AD623 | 11 | 24.4% |
|  | Homebrew | 12 | 26.7% |
|  | Others | 6 | 13.3% |

Supplementary Table 2: Assay configurations of the 45 *BCR-ABL1* tests.

1. **Auxiliary-pick-regression analysis to determine linearity against sample input**

In Study 1, linear regression analysis was performed to determine whether an assay could produce similar %BCR-ABL1 results against different sample inputs. To allow for the possibility that the highest or lowest sample input level might be outside of the assay’s linear dynamic range, data from either one of these two input levels might be omitted from the linear regression analysis. To determine whether it was statiscally appropriate to exclude one of the data points, the auxiliary-pick-regression method was used, in which Akaike information criterion (AIC) or Bayesian information criterion (BIC) were calculated for each assay as followed:


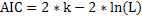


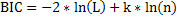


Where k was the number of estimated [parameters](https://en.wikipedia.org/wiki/Parameter) in the model, L was the maximum value of the [likelihood function](https://en.wikipedia.org/wiki/Likelihood_function) for the model, and n was the number of data points.

The AIC or BIC values from the 3 models were then compared: full model (include all data), the model excluding the lowest input level and the model excluding the highest input level. The model with the smallest AIC or BIC was chosen as the best fitted model and used to evaluate the assay’s linearity against different samples inputs. The results are shown in Supplementary Table 3 and 4. A graphical representation of results from each assay was shown in Supplementary Figure 2 and 3.

| Assay | Data Exclusion | Trend in Study 1 at MR^1^ | |
| --- | --- | --- | --- |
|  |  | Before Exclusion | After Exclusion |
| Assay 01 | All | Increasing | Increasing |
| Assay 02 | Exclude lowest input | No trend | No trend |
| Assay 03 | All | Increasing | Increasing |
| Assay 04 | Exclude lowest input | No trend | No trend |
| Assay 05 | Exclude highest input | Decreasing | Decreasing |
| Assay 06 | All | No trend | No trend |
| Assay 07 | Exclude highest input | Increasing | Increasing |
| Assay 08 | All | Decreasing | Decreasing |
| Assay 09 | All | Increasing | Increasing |
| Assay 10 | Exclude lowest input | No trend | No trend |
| Assay 11 | Exclude highest input | No trend | No trend |
| Assay 12 | Exclude lowest input | No trend | No trend |
| Assay 13 | Exclude lowest input | Decreasing | Decreasing |
| Assay 14 | Exclude lowest input | Increasing | Increasing |
| Assay 15 | Exclude highest input | Increasing | No trend |
| Assay 16 | All | No trend | No trend |
| Assay 17 | All | Increasing | Increasing |
| Assay 18 | Exclude highest input | No trend | Increasing |
| Assay 19 | Exclude highest input | Decreasing | Decreasing |
| Assay 20 | All | No trend | No trend |
| Assay 21 | Exclude highest input | Decreasing | Decreasing |
| Assay 22 | Exclude highest input | No trend | No trend |
| Assay 23 | All | Decreasing | Decreasing |
| Assay 24 | All | Decreasing | Decreasing |
| Assay 25 | All | No trend | No trend |
| Assay 26 | Exclude highest input | Decreasing | Decreasing |
| Assay 28 | Exclude highest input | Decreasing | Decreasing |
| Assay 30 | Exclude lowest input | Decreasing | Decreasing |
| Assay 31 | All | Decreasing | Decreasing |
| Assay 32 | Exclude highest input | Decreasing | No trend |
| Assay 33 | All | No trend | No trend |
| Assay 34 | Exclude highest input | Decreasing | No trend |
| Assay 35 | All | No trend | No trend |
| Assay 36 | Exclude highest input | No trend | No trend |
| Assay 37 | All | No trend | No trend |
| Assay 38 | Exclude highest input | Increasing | Increasing |
| Assay 39 | Exclude lowest input | No trend | No trend |
| Assay 40 | Exclude lowest input | Increasing | Increasing |
| Assay 41 | All | No trend | No trend |
| Assay 42 | Exclude highest input | Decreasing | No trend |
| Assay 43 | Exclude lowest input | Increasing | Increasing |
| Assay 44 | Exclude lowest input | Decreasing | No trend |
| Assay 45 | All | Decreasing | Decreasing |

Supplementary Table 3: Study 1 results for Vial A (MR^1^).

| **Assay** | **Exclusion** | **Trend in Study 1 at MR^3^** | |
| --- | --- | --- | --- |
|  |  | **Before Exclusion** | **After Exclusion** |
| Assay 01 | Exclude lowest input | Increasing | Increasing |
| Assay 02 | Exclude lowest input | No trend | No trend |
| Assay 03 | All | No trend | No trend |
| Assay 04 | Exclude lowest input | No trend | No trend |
| Assay 05 | Exclude lowest input | Decreasing | Decreasing |
| Assay 06 | Exclude highest input | Decreasing | No trend |
| Assay 07 | Exclude highest input | Increasing | Increasing |
| Assay 08 | All | No trend | No trend |
| Assay 09 | Exclude lowest input | Increasing | Increasing |
| Assay 10 | Exclude lowest input | No trend | Decreasing |
| Assay 11 | Exclude lowest input | No trend | No trend |
| Assay 12 | Exclude lowest input | No trend | No trend |
| Assay 13 | Exclude lowest input | Decreasing | No trend |
| Assay 14 | Exclude highest input | Increasing | No trend |
| Assay 15 | All | Increasing | Increasing |
| Assay 16 | All | No trend | No trend |
| Assay 17 | Exclude lowest input | Increasing | No trend |
| Assay 18 | Exclude lowest input | No trend | Decreasing |
| Assay 19 | Exclude highest input | Decreasing | Decreasing |
| Assay 20 | Exclude lowest input | Increasing | No trend |
| Assay 21 | All | Decreasing | Decreasing |
| Assay 22 | Exclude lowest input | No trend | No trend |
| Assay 23 | Exclude highest input | Decreasing | Decreasing |
| Assay 24 | All | No trend | No trend |
| Assay 26 | All | Decreasing | Decreasing |
| Assay 27 | All | No trend | No trend |
| Assay 28 | All | Decreasing | Decreasing |
| Assay 29 | All | Increasing | Increasing |
| Assay 30 | All | Decreasing | Decreasing |
| Assay 31 | Exclude lowest input | No trend | Increasing |
| Assay 32 | All | Decreasing | Decreasing |
| Assay 33 | All | No trend | No trend |
| Assay 34 | All | No trend | No trend |
| Assay 35 | All | No trend | No trend |
| Assay 36 | All | No trend | No trend |
| Assay 37 | Exclude highest input | No trend | No trend |
| Assay 38 | Exclude highest input | Increasing | No trend |
| Assay 39 | All | No trend | No trend |
| Assay 40 | All | Increasing | Increasing |
| Assay 41 | All | No trend | No trend |
| Assay 42 | Exclude highest input | Decreasing | No trend |
| Assay 43 | All | Increasing | Increasing |
| Assay 44 | Exclude lowest input | Decreasing | No trend |
| Assay 45 | Exclude highest input | Decreasing | No trend |

Supplementary Table 4: Study 1 results for Vial C (MR^3^).


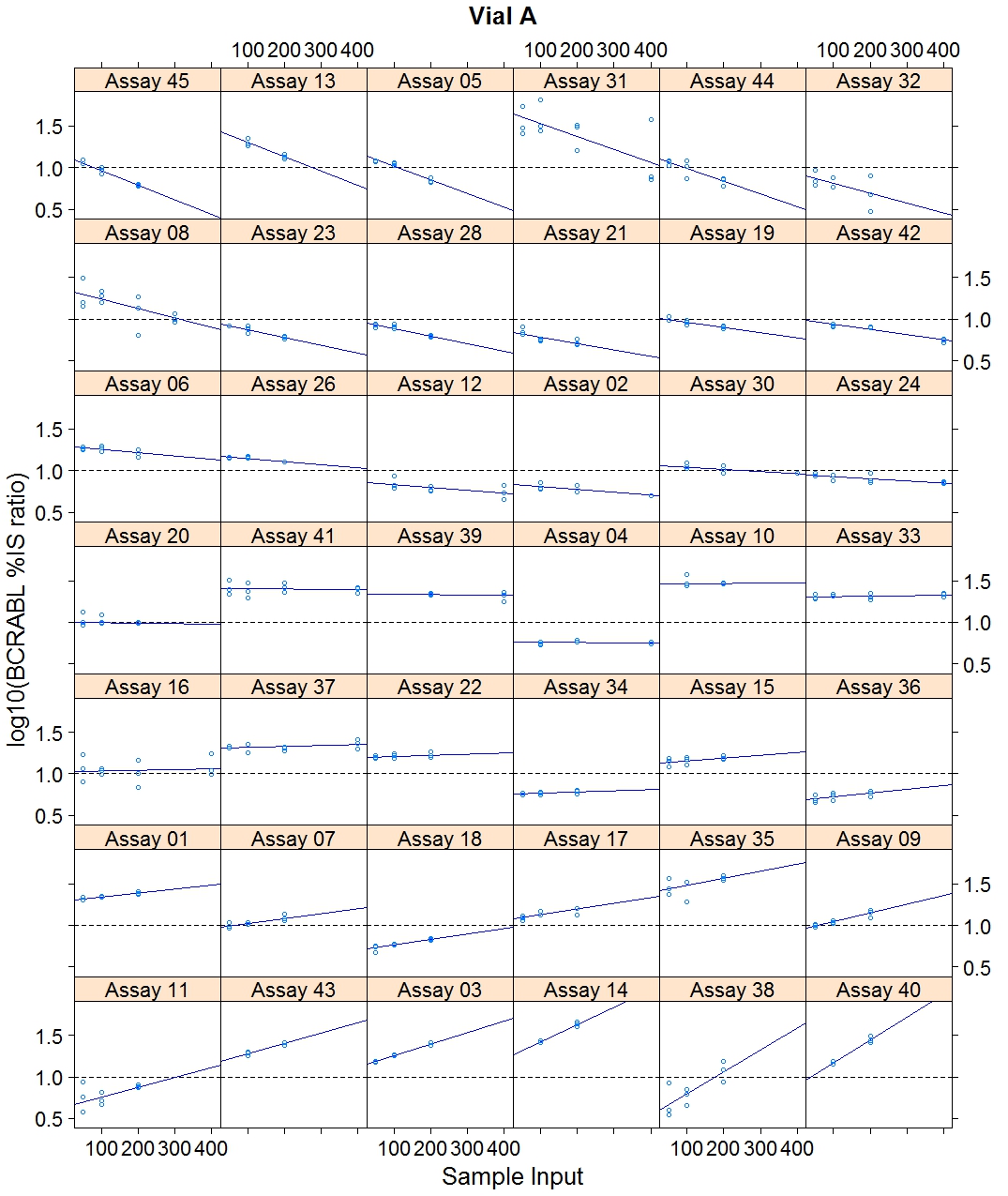


Supplementary Figure 2: Study 1 results from each *BCR-ABL1* assay for Vial A (MR^1^).


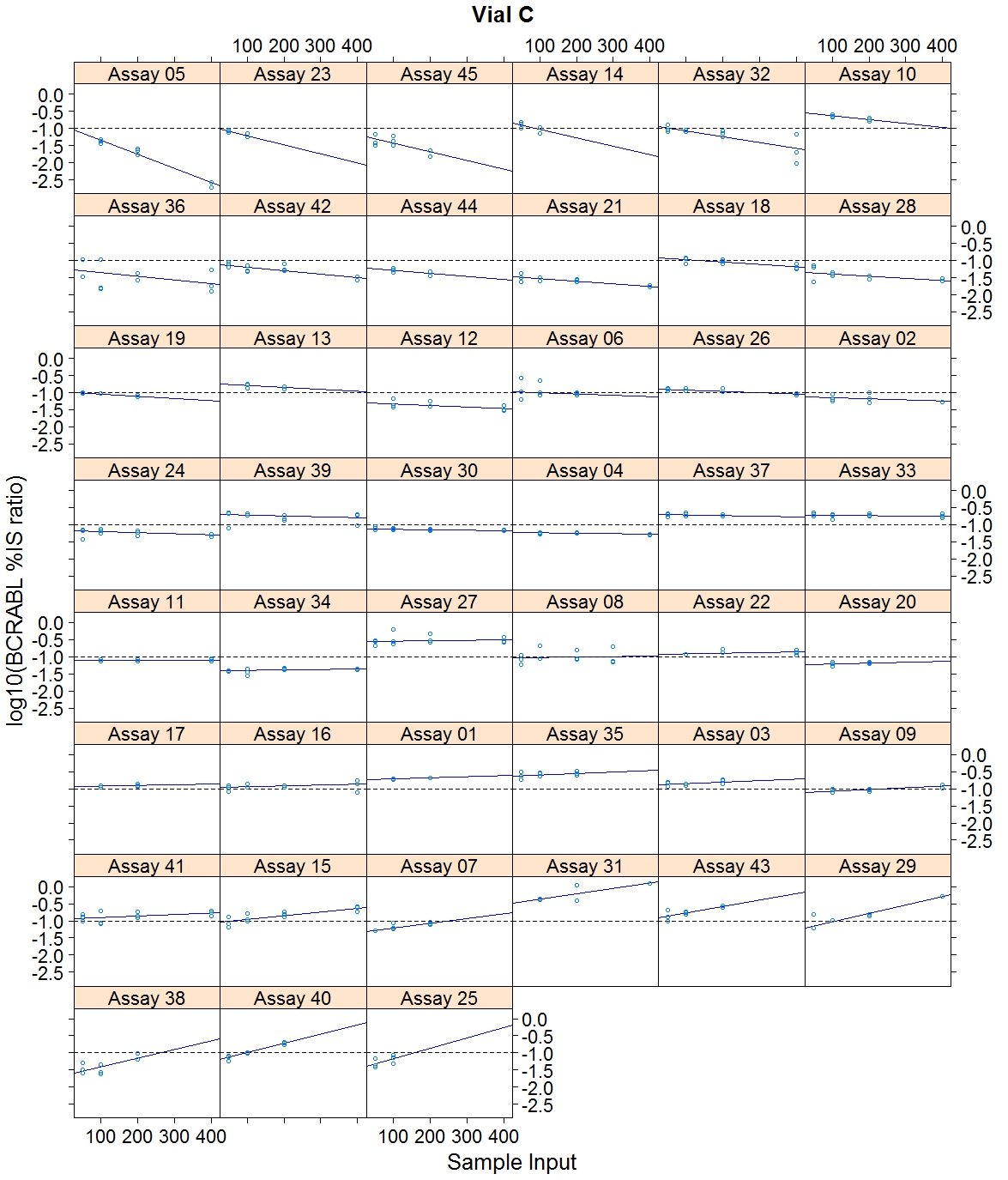


Supplementary Figure 3: : Study 1 results from each *BCR-ABL1* assay for Vial C (MR^3^).

1. **Calculation of PCR efficiency**

The slope of the standard curve for each *BCR-ABL1* and reference gene assay was calculated using Microsoft Excel with the Ct values on the y-axis and log sample input on the x-axis. Note that 2 standard curves were run by each laboratory, one using Vial A (MR^1^) and one using Vial C (MR^3^) of the secondary panel. Separate slopes were calculated for each sample for both the *BCR-ABL1* and reference gene assay. The PCR efficiency for each assay was subsequently calculated using the following formula:^3^

Efficiency =
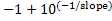


1. **Optimal sample input analysis**

Assay-specific optimal sample input for the secondary panel was calculated based on the self-reported average chronic myeloid leukemia (CML) patient sample input for each assay, in terms of reference gene copy number. The cell lines used to manufacture the secondary panel contained higher copy number of *BCR-ABL1* and reference genes per ng of RNA compared to typical CML patient samples. Thus, the optimal sample input for the secondary panel would be the amount of RNA that would introduce similar number of reference gene copies per PCR reaction compared to an average CML patient sample for the specific assay. To calculate the optimal sample input, linear regression analysis was performed using each assay’s data from Study 1, with the reference gene copy number on the y-axis and the ng sample input on the x-axis. The optimal sample input of the secondary panel (“x” in the linear regression analysis) was then calculated by entering the laboratory’s self-reported average CML patient sample reference gene copy number as the “y” value in the linear regression analysis. The average value taken from the Vial A (MR^1^) and Vial C (MR^3^) data for each assay was used as the final optimal sample input of the secondary panel for that assay.

The fold-difference in sample input between CML patient samples and the secondary panel for all participating laboratories is listed in Supplementary Table 5. Using robust regression analysis and normal probability plot, the results from Lab 07 and Lab 23 were identified as outliers and excluded from the analysis (Supplementary Figure 4). A robust mean of 2.4-fold was calculated through robust regression. The robust regression is done by using M-estimator.^4^

| **Lab ID** | **Mean(Fold difference in patient vs cell sample input)** |
| --- | --- |
| Assay 25 | 0.18 |
| Assay 03 | 0.58 |
| Assay 14 | 0.69 |
| Assay 10 | 0.77 |
| Assay 44 | 0.83 |
| Assay 22 | 0.85 |
| Assay 13 | 0.99 |
| Assay 12 | 1.05 |
| Assay 43 | 1.19 |
| Assay 45 | 1.23 |
| Assay 17 | 1.32 |
| Assay 15 | 1.58 |
| Assay 20 | 1.59 |
| Assay 21 | 1.67 |
| Assay 04 | 1.76 |
| Assay 42 | 1.78 |
| Assay 11 | 1.89 |
| Assay 30 | 2.45 |
| Assay 24 | 2.50 |
| Assay 33 | 2.66 |
| Assay 18 | 2.72 |
| Assay 09 | 2.97 |
| Assay 41 | 3.17 |
| Assay 34 | 3.77 |
| Assay 39 | 3.88 |
| Assay 26 | 4.58 |
| Assay 28 | 4.78 |
| Assay 19 | 5.58 |
| Assay 16 | 5.80 |
| Assay 07 | 11.56 |
| Assay 23 | 48.26 |

Supplementary Table 5: Fold-difference in ng sample input between CML patient sample and the secondary panel for each assay.


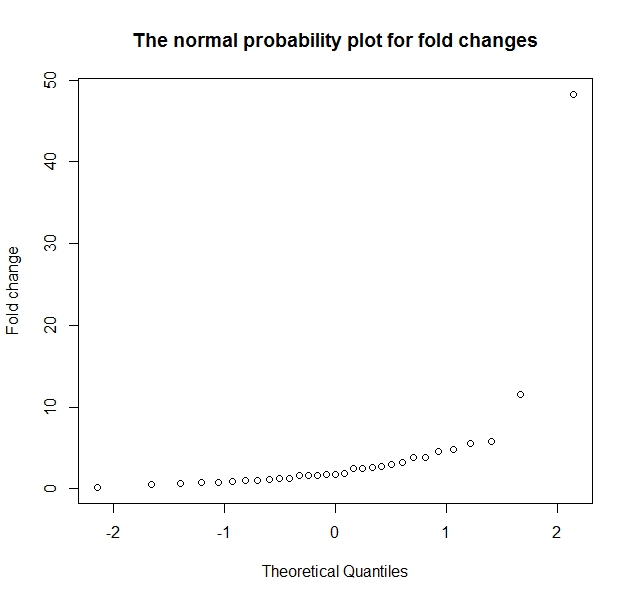


Supplementary Figure 4: Outlier check using robust regression analysis.

1. **Calculation of mean % BCR-ABL1 from all labs**

Robust mean value of the %BCR-ABL1 from all 45 tests for each panel member by reference gene was calculated through robust regression in R package called “MASS”. The robust regression was performed using M-estimator^4^. Graphical representations of results from all 45 assays at each level were shown in Supplementary Figure 5a-e.

(a) Results from Vial A (MR^1^).


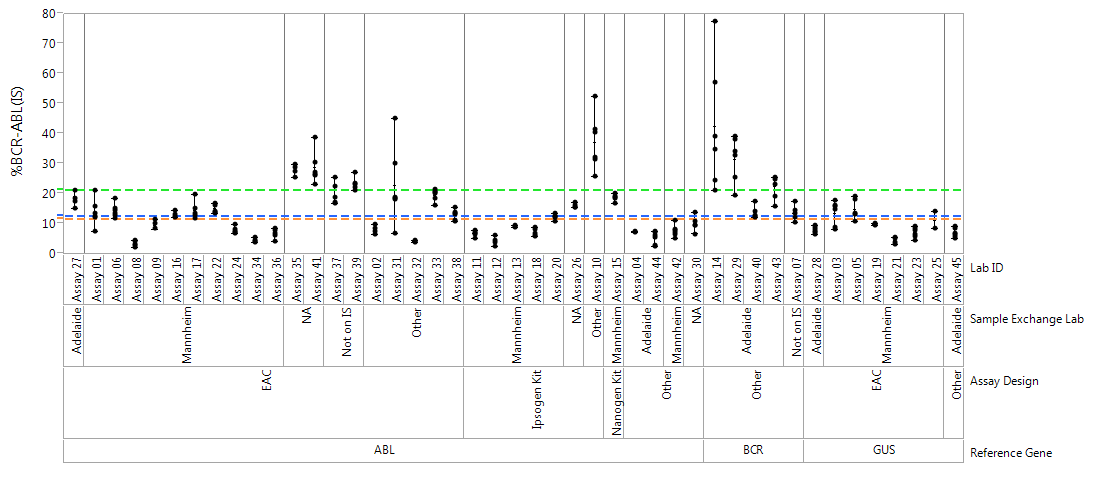


(b) Results from Vial B (MR^2^).


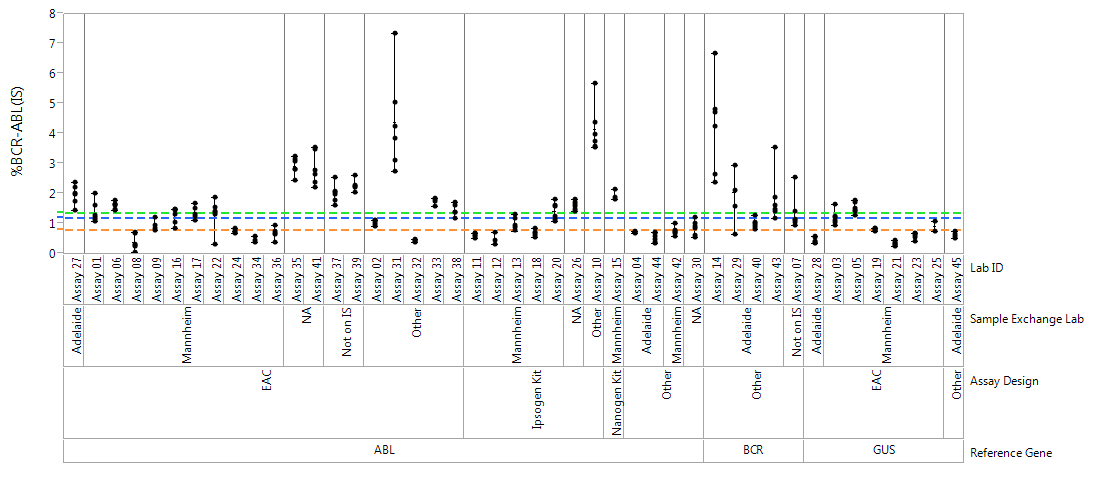


(c) Results from Vial C (MR^3^).


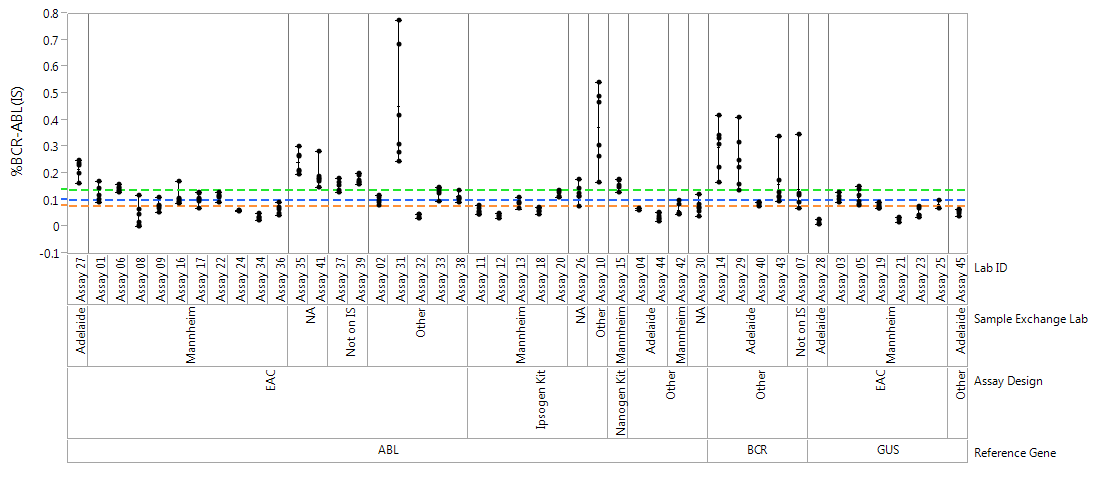


(d) Results from Vial D (MR^4^).


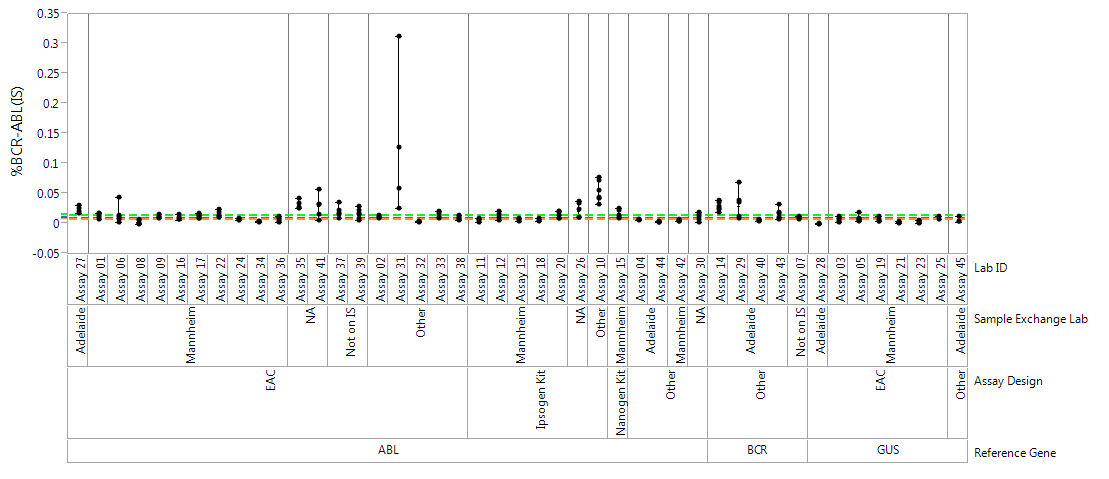


(e) Results from Vial E (MR^4.5^).


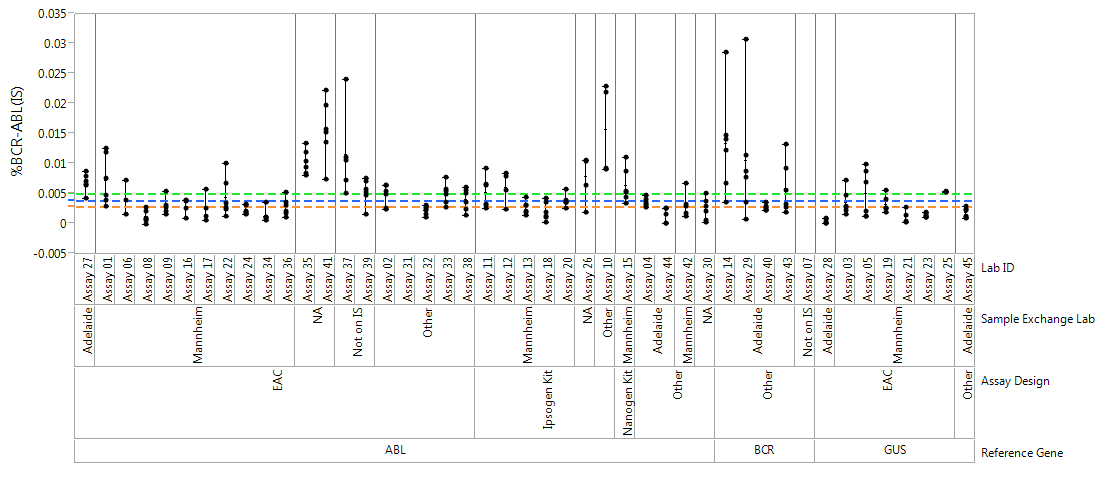


Supplementary Figure 5: %BCR-ABL1 results from all assays for each level of the secondary panel, including (a) Vial A at MR^1^, (b) Vial B at MR^2^, (c) Vial C at MR^3^, (d) Vial D at MR^4^, and (e) Vial E at MR^4.5^. Dotted lines in the graphs indicate the assigned %BCR-ABL^IS^ values of the secondary panel (*BCR*: green, *ABL1*: blue, *GUSB*: orange).

1. **Correlation between assay performance and different assay characteristics**

To quantify the assay performance for each assay, we developed a scoring method that captured information on the IS accuracy, precision and detection rate of each assay. A performance score was calculated for each assay using the following formula:


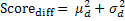


Bayesian model averaging is a way of taking account of uncertainty about the interested variables to be included in linear regression. Here Bayesian model averaging regression analysis was performed by using the R package called “BMA” to assess whether a statistically significant correlation can be observed between the scores and parameters including reference gene, RNA extraction method and usage of commercial kit versus laboratory-developed test (LDT).

1. **IS conversion factor (CF) comparison**

The following calculation was performed to determine the sample exchange CF/secondary panel CF ratio that would lead to < 0.2 log difference in the resulting %BCR-ABL1 values:

Assuming the laboratory’s current conversion factor (CF) is *A* and the CF calculated from the secondary panel is B, for any *BCR-ABL1* ratio *p*, the
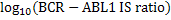
 is,


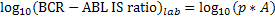


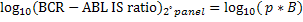


The difference of
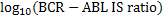
 between the laboratory’s current CF and the secondary panel CF is,


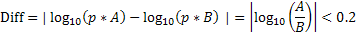


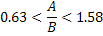


The ratio between the lab’s current CF and CF calculated from the secondary panel should be between 0.63 and 1.58 in order to have the MR difference between the sample exchange CF and secondary panel CF < 0.2 log. If the ratio is closer to 1 which means there is minor difference.

1. **RT-ddPCR assays development and analytical validation**

Primers and probes for RT-ddPCR assays against *BCR-ABL1*, *ABL1*, *BCR,* and *GUSB* listed in Table 1 in the main manuscript were designed using Primer Express software (Applied Biosystems, Foster City, California, USA). The analytical validation studies were performed for all 4 RT-ddPCR assays to assess their Limit of Blank (LoB), Limit of Detection (LoD), linearity and precision. The validation studies were designed following recommendations from Minimum Information for Publication of Quantitative Digital PCR Experiments (MIQE)^5^and Clinical and Laboratory Standards Institute (CLSI) guidelines,^6-8^ as well as industry best practices.^9^

1. LoB
2. Samples

- For *BCR-ABL1* assay only: RNA was isolated from K_2_EDTA blood samples from 2 healthy donors using the Maxwell 16 LEV simplyRNA Blood Kit (Promega, Madison, Wisconsin, USA). cDNA was generated from the pooled RNA using High Capacity cDNA Reverse Transcription Kit (Applied Biosystems, Foster City, California, USA). 990 ng of healthy donor K_2_EDTA blood cDNA was tested per reaction well. RNA extracted from K562 cells was used as a positive control on each plate to ensure that the RT-ddPCR reactions were successful.
- For *BCR-ABL1, ABL1, BCR,* and *GUSB* assays: Reverse transcription (RT) reactions were conducted using nuclease-free water (Ambion, Austin, Texas, USA) as no template control (NTC) samples with the High Capacity cDNA Reverse Transcription Kit (Applied Biosystems). Panel member E of the secondary reference panel or RNA extracted from K562 cells were used as a positive control on each plate to ensure that the RT-ddPCR reactions were successful.

1. Experimental Procedures

- For *BCR-ABL1* assay: A total of 792 replicates of healthy donor K_2_EDTA blood cDNA were run on 9 different plates. In addition, a total of 60 replicates of NTC were run on 2 different plates.
- For *ABL1* assay: A total of 60 replicates of NTC were run on 2 different plates.
- For *BCR* and *GUSB* assays: A total 160 replicates of NTC were run on 4 different plates.

ddPCR was performed using 2X ddPCR Supermix (Bio-Rad, Hercules, California, USA) on either the QX-100 or QX-200 ddPCR system (Bio-Rad). The QX-100 and QX-200 systems were previously determined to yield equivalent results (data not shown). Any wells with < 9025 accepted droplets were considered invalid, following the manufacturer’s recommendations. The 95^th^ percentile of reported results was established as the LoB for each assay.

1. Results

The LoB is defined as the highest measurement result that is likely to be observed when a blank sample is tested.^8^ In the case of ddPCR, it can be considered as the number of false positive droplets that may be observed in a blank sample. To determine the LoB of each ddPCR assay, the number of replicates that detected false positive droplets was recorded and shown in Supplementary Table 6.

| # Positive droplets detected | Number of RT-ddPCR replicates | | | | |
| --- | --- | --- | --- | --- | --- |
|  | *BCR-ABL1* assay | | *ABL1* assay | *BCR* assay | *GUSB* assay |
|  | Donor blood | NTC | NTC | NTC | NTC |
| 0 | 645 | 55 | 35 | 147 | 154 |
| 1 | 54 | 3 | 18 | 1 | 4 |
| 2 | 7 | 1 | 5 | 0 | 1 |
| 3 | 2 | 0 | 0 | 0 | 0 |
| 4 | 0 | 0 | 0 | 0 | 0 |
| 5 | 0 | 0 | 1 | 0 | 0 |
| 6 | 0 | 0 | 1 | 0 | 0 |
| 7 | 0 | 0 | 0 | 0 | 0 |
| 8 | 1 | 0 | 0 | 0 | 0 |
| 9 | 0 | 0 | 0 | 0 | 1 |
| Invalid Well | 83 | 1 | 0 | 12 | 0 |
| Total Valid Well | 709 | 59 | 60 | 148 | 160 |

Supplementary Table 6: Number of positive droplets detected for each ddPCR assay in the LoB study.

- For *BCR-ABL1* assay (single well): For the healthy donor blood cDNA, a total of 709 valid wells were run. 95% of 709 wells equals 673.55 wells. Since a total of 699 wells reported ≤ 1 positive droplets, the LoB was 1 positive droplet. For the NTC samples tested, a total of 59 valid wells were run. 95% of 59 wells equals 56.05 wells. Since a total of 58 wells reported ≤ 1 positive droplet, the LoB was also 1 positive droplet. Based on both sets of results, the calculated LoB for the *BCR-ABL1* assay in single well analysis was 1 positive droplet, indicating that only samples that reported > 1 positive droplet will be considered a positive sample.
- For *BCR-ABL1* assay (merged well): ddPCR allowed the merging of multiple reaction wells to increase the total sample input, and thus the sensitivity, of an assay. LoB analysis was performed by merging different numbers of wells using data from the 709 wells. The LOB was calculated to be 2 positive droplets for 4- and 6-merged wells, 3 positive droplets for 8-merged wells, and 4 positive droplets for 10-merged wells.
- For *ABL1* assay: A total of 60 valid wells were run. 95% of 60 wells equals 57 wells. Since a total of 58 wells reported ≤ 2 positive droplets, the LoB was 2 positive droplets. Thus, only samples that reported > 2 positive droplet will be considered a positive sample.
- For *BCR* assay: A total of 148 valid wells were run. 95% of 60 wells equals 140.6 wells. Since a total of 147 wells reported 0 positive droplets, the LoB was 0 positive droplets. Thus, samples that reported > 0 positive droplet will be considered a positive sample.
- For *GUSB* assay: A total of 160 valid wells were run. 95% of 160 wells equals 152 wells. Since a total of 154 wells reported 0 positive droplets, the LoB was 0 positive droplets. Thus, samples that reported > 0 positive droplet will be considered a positive sample.

1. Conclusions

The LoB for the *BCR-ABL1* assay (single well analysis) was 1 positive droplet, indicating that only samples with > 1 positive droplets can be considered positive. The LoB increased to 2 positive droplets in 4- and 6-merged well analysis for *BCR-ABL1*, 3 positive droplets in 8-merged well analysis, and 4 positive droplets in 10-merged well analysis. The LoB for the *ABL1* assay was 2 positive droplets, and the LoB for the *BCR* and *GUSB* assays was 0 positive droplet.

1. LoD, linearity and precision

The LoD, linearity and precision of the *BCR-ABL1*, *ABL1*, *BCR,* and *GUSB* ddPCR assays were assessed using data from the same studies.

1. Samples

- For *BCR-ABL1* and *ABL1* assays: RNA was isolated from K562 cells (ATCC CCL-243) using the RNeasy® Mini Kit (Qiagen). cDNA was generated using High Capacity cDNA Reverse Transcription Kit (Applied Biosystems).
- For *BCR-ABL1* assay only: To establish the LoD of the *BCR-ABL1* assay in CML patient samples, a contrived patient sample panel was created by serially diluting RNA extracted from a CML patient’s K_2_EDTA blood sample and RNA extracted from a donor K_2_EDTA blood sample. The panel consisted of 4 *BCR-ABL1* levels targeting MR^3^, MR^4^, MR^4.5^, and MR^5^.
- For *BCR* and *GUSB* assays: RNA was isolated from panel member E of the secondary reference panel using the RNeasy® Mini Kit (Qiagen). cDNA was generated using High Capacity cDNA Reverse Transcription Kit (Applied Biosystems).

1. Experimental Procedures

The following studies were performed to assess the LoD, linearity, and precision of the 4 ddPCR assays. The sample input range was selected so that the highest input would result in ~30 000 copies per reaction well, as the number of droplets per reaction for the Bio-Rad QX-100 and QX-200 systems was ~15 000. The lowest input for *BCR-ABL1* was targeted to be ~1 copy per well, whereas the lowest input for the reference genes *ABL1, BCR,* and *GUSB* was targeted to be ~10 copies per well, since the reference genes are typically present in much higher abundance than *BCR-ABL1* in CML patient samples.

- For *BCR-ABL1* assay (cell line cDNA): To generate a standard curve, different sample inputs of K562 cell cDNA were used for the ddPCR reactions, including 35 ng, 1.5 ng, 7.5 pg, 3.75 pg, 1.875 pg, and 0.9375 pg. A total of 30 replicates at each input level were run for each assay on 2 separate plates.
- For *BCR-ABL1* assay (contrived CML patient sample): 843.75 ng of cDNA of each sample in the contrived CML patient panel (MR^3^, MR^4^, MR^4.5^, and MR^5^) was used per ddPCR reaction. A total of 100 replicates of each sample were run on 5 different plates.
- For *ABL1* assay: To generate a standard curve, different sample inputs of K562 cell cDNA were used for the ddPCR reactions, including 22 ng, 1.5 ng, 0.15 ng, and 0.015 ng. A total of 36 replicates at each input level were run for each assay on 2 separate plates.
- For *BCR* and *GUSB* assays: To generate a standard curve, different sample inputs of panel member E cDNA were used for the ddPCR reactions, including 30 ng, 3 ng, 0.3 ng, 0.03 ng, and 0.01 ng. A total of 48 replicates at each input level were run for each assay on 6 separate plates.

Nuclease-free water (Ambion) was included on every plate as an NTC. ddPCR was performed using 2X ddPCR Supermix (Bio-Rad) on either the QX-100 or QX-200 ddPCR system (Bio-Rad). The QX-100 and QX-200 systems were previously determined to yield equivalent results (data not shown). Any wells with < 9025 accepted droplets were considered invalid, following the manufacturer’s recommendations.

1. Results

*LoD*

The LoD of an assay is the sample input level that can be successfully detected by the assay over 95% of the time.^8^ In this study, we calculated the LoD for ddPCR as the number of copies per reaction well.

- For *BCR-ABL1* assay: The detection rate of the *BCR-ABL1* assay at different *BCR-ABL1* level of the contrived CML patient sample panel cDNA was listed in Supplementary Table 7. For single well analysis, the detection rate at MR^4.5^ (9.2 copies per well) was 97%, whereas the detection rate at MR^5^ (4.4 copies per well) was 65%. Thus, the LoD of the *BCR-ABL1* assay in single well analysis was between 9.2 and 4.4 copies per reaction well. However, when 5 ddPCR wells were analyzed together in merged well analysis, the detection rate was 100% even at MR^5^, with an average of 16.4 copies per 5 wells. Thus, the LoD of the *BCR-ABL1* assay in 5-merged well analysis was below 16.4 copies and MR^5^.

| Sample | Single Well Analysis | | | 5-Merged Well Analysis | | |
| --- | --- | --- | --- | --- | --- | --- |
|  | Mean copy per well | Replicates detected | Detection rate | Mean copy per 5-well | Replicates detected | Detection rate |
| A (MR^3^) | 290.4 | 100/100 | 100% | 1452.0 | 20/20 | 100% |
| B (MR^4^) | 28.1 | 100/100 | 100% | 140.7 | 20/20 | 100% |
| C (MR^4.5^) | 9.2 | 97/100 | 97% | 44.6 | 20/20 | 100% |
| D (MR^5^) | 4.4 | 65/100 | 65% | 16.4 | 20/20 | 100% |

Supplementary Table 7: Detection rate of the *BCR-ABL1* assay.

- For *ABL1* assay: The detection rate of the *ABL1* assay was 100% at all tested sample input level of the K562 cDNA, including 22 ng, 1.5 ng, 0.15 ng, and 0.015 ng. The mean copy number per well at 0.015 ng was 32.2. Thus, the LoD of the *ABL1* assay was < 32.2 copies per reaction well.
- For *BCR* assay: The detection rate of the *BCR* assay was 100% at all tested sample input level of the panel member E cDNA, including 30 ng, 3 ng, 0.3 ng, 0.03 ng, and 0.01 ng. The mean copy number per well at 0.01 ng was 10.3. Thus, the LoD of the *BCR* assay was < 10.3 copies per reaction well.
- For *GUSB* assay: The detection rate of the *GUSB* assay was 100% at all tested sample input level of the panel member E cDNA, including 30 ng, 3 ng, 0.3 ng, 0.03 ng, and 0.01 ng. The mean copy number per well at 0.01 ng was 11.2. Thus, the LoD of the *GUSB* assay was < 11.2 copies per reaction well.

*Precision*

The precision of the ddPCR assays was calculated in terms of the percent coefficient of variation (%CV) of the measured copy per reaction well (Supplementary Table 8).^7^ For all 4 ddPCR assays, the %CV was directly correlated with the amount of sample input. At higher sample inputs that resulted in at least a few hundred copies per well, the %CV was under 10%. At fewer than 10 copies per well, the %CV increased dramatically to > 50%. Thus, increased sample input could improve precision in ddPCR. In addition, precision can be improved by performing merged well analysis. In the contrived CML patient sample panel, by merging data from 5 wells, the %CV decreasd by approximately half from MR^3^ to MR^5^ (Supplementary Table 9).

| Assay | Sample | Sample Input (ng) | N | Mean Copy Per Well | %CV |
| --- | --- | --- | --- | --- | --- |
| *BCR-ABL1* | K562 cDNA | 35 | 30 | 62800.0 | 3.0 |
|  |  | 1.5 | 30 | 2530.0 | 6.6 |
|  |  | 0.0075 | 30 | 14.6 | 41.5 |
|  |  | 0.00375 | 28 | 6.6 | 53.9 |
|  |  | 0.00188 | 30 | 4.7 | 60.9 |
|  |  | 0.00094 | 30 | 2.8 | 75.0 |
| *ABL1* | K562 cDNA | 22 | 35 | 56188.6 | 3.1 |
|  |  | 1.5 | 36 | 3546.7 | 3.3 |
|  |  | 0.15 | 35 | 348.2 | 9.4 |
|  |  | 0.015 | 36 | 32.2 | 18.4 |
| *BCR* | Panel member E cDNA | 30 | 48 | 28845.8 | 5.7 |
|  |  | 3 | 48 | 2895.0 | 7.9 |
|  |  | 0.3 | 48 | 287.5 | 9.1 |
|  |  | 0.03 | 48 | 34.4 | 25.0 |
|  |  | 0.01 | 47 | 10.3 | 48.9 |
| *GUSB* | Panel member E cDNA | 30 | 47 | 34897.9 | 6.6 |
|  |  | 3 | 48 | 3452.9 | 7.2 |
|  |  | 0.3 | 48 | 350.4 | 9.4 |
|  |  | 0.03 | 47 | 31.2 | 24.1 |
|  |  | 0.01 | 47 | 11.2 | 32.6 |

Supplementary Table 8: Precision of the *BCR-ABL1*, *ABL1*, *BCR* and *GUSB* RT-ddPCR assays in terms of %CV.

| Assay | Sample | Sample Input (ng) | Analysis | *BCR-ABL1* Level | N | Mean Copy Per Well | %CV |
| --- | --- | --- | --- | --- | --- | --- | --- |
| *BCR-ABL1* | Contrived CML Patient Sample Panel | 843.75 ng | Single well | MR^3^ | 100 | 290.4 | 7.3 |
|  |  |  |  | MR^4^ |  | 28.1 | 24.5 |
|  |  |  |  | MR^4.5^ |  | 9.2 | 39.7 |
|  |  |  |  | MR^5^ |  | 4.4 | 38.9 |
|  |  |  | 5-merged wells | MR^3^ |  | 1452.0 | 4.3 |
|  |  |  |  | MR^4^ |  | 140.7 | 11.6 |
|  |  |  |  | MR^4.5^ |  | 44.6 | 20.3 |
|  |  |  |  | MR^5^ |  | 16.4 | 33.8 |

Supplementary Table 9: Precision of the *BCR-ABL1* RT-ddPCR assay in terms of %CV.

To assess precision in terms of %BCR-ABL1, the SD of log(%BCR-ABL1) for each RT-ddPCR assay was calculated using data from the IS calibration of the secondary panel against the WHO panel (n=36-40 for MR^1^ – MR^4^, n=75-77 for MR^4.5^) (Figure 1 and Table 2a). The calculated SD for all 3 assays at each *BCR-ABL1* level of the secondary panel, using single well analysis only, were well under 0.25 log (Supplementary Table 10), indicating that all 3 RT-ddPCR assays had excellent precision in terms of %BCR-ABL1 measurements, even at MR^4.5^.

| Secondary Panel Member | Standard Deviation of log(%BCR-ABL1) | | |
| --- | --- | --- | --- |
|  | *BCR-ABL1/ABL1* | *BCR-ABL1/BCR* | *BCR-ABL1/GUS* |
| A (MR^1^) | 0.022 | 0.039 | 0.024 |
| B (MR^2^) | 0.025 | 0.043 | 0.039 |
| C (MR^3^) | 0.034 | 0.046 | 0.041 |
| D (MR^4^) | 0.110 | 0.090 | 0.096 |
| E (MR^4.5^) | 0.180 | 0.190 | 0.170 |

Supplementary Table 10: Precision of the RT-ddPCR assays in terms of log(%BCR-ABL1).

*Linearity*

To evaluate assay linearity, log_10_ concentration was plotted against log_10_ sample input for each assay and linear regression analysis was performed.^6^ The resulting R^2^ value was 0.98 for the *BCR-ABL1* assay, 0.99 for *ABL1*, 0.99 for *BCR,* and 1.00 for *GUSB*, indicating excellent linearity for all assays over the measured range of ~10 to ~30 000 target molecules per reaction well for *ABL1*, *BCR,* and *GUS*, and ~1 to ~30 000 copies for *BCR-ABL1*.

1. Conclusions

The LoD was between 9.4 and 4.4 copies per ddPCR reaction for the *BCR-ABL1* assay in single well analysis, < 16.4 copies per 5-well in the *BCR-ABL1* 5-merged well analysis, < 32.2 copies per reaction for the *ABL1* assay, < 10.3 copies per reaction for the *BCR* assay and < 11.2 copies per reaction for the *GUSB* assay. The precision of all 4 RT-ddPCR assays was correlated with sample input, with the %CV of each assay being well under 10% when at least a few hundred copies were present per reaction well. In terms of %BCR-ABL1, the SD of log(%BCR-ABL1) for all 3 ddPCR assays using single well analysis were well under 0.25 log, indicating excellent precision even down to MR^4.5^. The linearity of all four ddPCR assays were excellent over the sample input range tested (~1 to ~30 000 copies for *BCR-ABL1*, ~10 to ~30 000 copies for *ABL1*, *BCR,* and *GUS*) , with an R^2^ of > 0.98.

**REFERENCES**

1. White HE, Matejtschuk P, Rigsby P, Gabert J, Lin F, Lynn Wang Y*, et al.* Establishment of the first World Health Organization International Genetic Reference Panel for quantitation of BCR-ABL mRNA. *Blood* 2010 Nov 25; **116**(22)**:** e111-117.

2. Thiers RE, Wu GT, Reed AH, Oliver LK. Sample stability: a suggested definition and method of determination. *Clinical chemistry* 1976 Feb; **22**(2)**:** 176-183.

3. Rasmussen R. Quantification on the LightCycler. In: Meuer S, Wittwer, C., Nakagawara, K. (ed). *Rapid Cycle Real-time PCR, Methods and Applications.* . Spinger Press: Heidelberg, 2001, pp 21-34.

4. Fox J. Robust regression. *An R and S-Plus companion to applied regression* 2002.

5. Huggett JF, Foy CA, Benes V, Emslie K, Garson JA, Haynes R*, et al.* The digital MIQE guidelines: Minimum Information for Publication of Quantitative Digital PCR Experiments. *Clinical chemistry* 2013 Jun; **59**(6)**:** 892-902.

6. Clinical and Laboratory Standards Institute. Evaluation of the linearity of quantitative measurement procedures: a statistical approach; approved guideline. Clinical and Laboratory Standards Institute, Wayne, PA; 2003.

7. Clinical and Laboratory Standards Institute. Evaluation of precision performance of quantitative measurement methods; approved guidelin, second edition. Clinical and Laboratory Standards Institute, Wayne, PA; 2004.

8. Clinical and Laboratory Standards Institute. Evaluation of detection capability for clinical laboratory measurement procedures; approved guideline—second edition. Clinical and Laboratory Standards Institute, Wayne, PA; 2012.

9. Jennings LJ, Smith FA, Halling KC, Persons DL, Kamel-Reid S, Molecular Oncology Resource Committee of the College of American P. Design and analytic validation of BCR-ABL1 quantitative reverse transcription polymerase chain reaction assay for monitoring minimal residual disease. *Archives of pathology & laboratory medicine* 2012 Jan; **136**(1)**:** 33-40.
